# Probabilistic modelling is superior to deterministic approaches in the human health risk assessment: an example from a tribal stretch in central India

**DOI:** 10.1038/s41598-023-45622-1

**Published:** 2023-11-07

**Authors:** Rajkumar Herojeet, Rakesh K. Dewangan, Pradeep K. Naik, Janak R. Verma

**Affiliations:** 1 Department of Environmental Studies, Post Graduate Government College, Sector-11, Chandigarh, 160011 India; 2 Central Ground Water Board, North Central Chhattisgarh Region, Ministry of Jal Shakti, Govt. of India, LK Corporates Tower, Dumartarai, Dhamtari Road, Raipur, 492015 India; 3 Present Address: Centre for Hydrological Sciences and Communication, Bhubaneswar, India

**Keywords:** Environmental sciences, Hydrology, Natural hazards, Diseases, Health care, Risk factors, Chemistry

## Abstract

This case drew national attention in 2018. About 100 people died and more than 300 hospitalized in a span of few years in a village of 1200 people in a tribal stretch in central India. Medical teams visiting the area reported severe renal failure and blamed the local eating and drinking habits as causative factors. This human health assessment based on geochemical investigations finds nitrate (NO_3_^−^) and fluoride (F^−^) pollution as well in village’s groundwater. Both deterministic and probabilistic techniques are employed to decipher the contamination pathways and extent of contamination. Source apportionments of NO_3_^−^ and F^−^ and their relationship with other ions in groundwater are carried out through chemometric modelling. Latent factors controlling the hydrogeochemistry of groundwater too are explored. While hazard quotients ($HQ$) of the chemical parameters ($HQ_{{{\text{NO}}_{3}^{ - } }}$ and $HQ_{{{\text{F}}^{ - } }}$) identify ingestion as the prominent pathway, the calculated risk certainty levels (RCL) of the hazard index (HI) values above unity are compared between the deterministic and probabilistic approaches. Deterministic model overestimates the HI values and magnify the contamination problems. Probabilistic model gives realistic results that stand at infants ($HI_{{{\text{NO}}_{3}^{ - } }}$ = 34.03%, $HI_{{{\text{F}}^{ - } }}$ = 24.17%) > children ($HI_{{{\text{NO}}_{3}^{ - } }}$ = 23.01%, $HI_{{{\text{F}}^{ - } }}$ = 10.56%) > teens ($HI_{{{\text{NO}}_{3}^{ - } }}$ = 13.17%, $HI_{{{\text{F}}^{ - } }}$ = 2.00%) > adults ($HI_{{{\text{NO}}_{3}^{ - } }}$ = 11.62%, $HI_{{{\text{F}}^{ - } }}$ = 1.25%). Geochemically, about 90% of the samples are controlled by rock-water interaction with Ca^2+^–Mg^2+^–HCO_3_^−^ (~ 56%) as the dominant hydrochemical facies. Chemometric modelling confirms Ca^2+^, Mg^2+^, HCO_3_^−^, F^−^, and SO_4_^2−^ to originate from geogenic sources, Cl^−^ and NO_3_^−^ from anthropogenic inputs and Na^+^ and K^+^ from mixed factors. The area needs treated groundwater for human consumption.

## Introduction

Globally, consumption of nitrate (NO_3_^−^) and fluoride (F^−^) contaminated groundwater is a serious concern due to their role in causing clinical diseases in humans^1–5^. Among the different inorganic forms of nitrogen (NO_3_^−^, NO_2_^−^ and NH_4_^+^) that exist in aquifers, NO_3_^−^ concentrations are higher than those of NO_2_^−^ and NH_4_^+^ due to their high solubility and mobility rates as well as higher stable oxidative state in water^6,7^. Both NO_2_^−^ and NH_4_^+^ are easily oxidized and converted to NO_3_^−^; thus, they have lower contents in groundwater^8^. Anthropogenic sources that contribute to excess NO_3_^−^ in groundwater system are overuse of N-fertilizers, excreta from livestock farms, municipal wastewater irrigation, runoff from urban and agricultural land, leaching from waste dumping sites and discharge of untreated sewage and industrial effluents^9–13^.

The natural sources of NO_3_^−^ in groundwater are the dissolution and oxidation of nitrogenous minerals in the sedimentary and metasedimentary rocks. The bedrock nitrogen minerals, such as nitraline, nitre, suhalite and tobelite, have three possible origins: organic matter, ammonium silicates and nitrate and ammonium salts^14^. Dissolution of these sources release ammonium from their crystal lattices into the soil horizon, the chemical form which can be easily assimilated by soil micro-organisms or get converted to NO_3_^−^ through the nitrification process for nitrogen fixation by leguminous plants^15–17^. On the other hand, the weathering process of organic nitrogen present in bedrocks are mineralized and converted to ammonium, which is readily used by the soil biota.

Recently, many workers highlighted the worldwide contamination of NO_3_^−^ in groundwater and its adverse effect on human health. Some locational examples are Loess Plateau, Northwest China^18^, Weining Plain, Northwest China^2^, Matanza-Riachuelo River Basin, Argentina^19^, Donsheng district, Inner Mongolia^20^, Catalan Region, Spain^21^, Gorveh-Dehgelan, Western Iran^22^, Shanmuganadhi river basin, southern India^23^, Jalandhar district, Punjab, India^24^, Panipat district, Haryana, India^25^, Nagpur, Western Maharashtra, India^26^, Gaya district, Bihar, India^8^, Tiruppur, Tamil Nadu, India^27^.

Water with NO_3_^−^ concentrations between 45 and 100 mg/L and above 100 mg/L are consumed daily for drinking purposes by ~ 118 million and ~ 108 million people, respectively, in India^28,29^. The common and predominant effect of excess NO_3_^−^ content (> 45 mg/L) in bottle-fed infants and children is Methemoglobinemia disease^24,30^. Almasri concludes that the clinical symptom of methemoglobinemia is normally encountered as body dehydration and gastrointestinal infections^31^. Further, the biochemical effects of NO_3_^−^ occur in the human body as follows: (a) NO_3_^−^ is converted to NO_2_^−^ under reducing conditions, (b) haemoglobin (Hb) combines with NO_2_^−^ to form methemoglobin, (c) the effect of methemoglobin reduces the oxygen supply in red blood cells and drops the oxygen level in the body and (d) higher rate of methemoglobin formations (> 10%) leads to the blueish colouration of the skin, known as a blue-baby syndrome (cyanosis)^4,32^. The prolonged exposure to high NO_3_^−^ content in water has other health risks, such as multiple sclerosis, nitrosamines and non-hodgkin lymphoma^33–35^.

The fluoride deposition on the earth's crust is approximately 0.32% and occurs mainly in rocks, such as granites and gneisses. Both natural and anthropogenic inputs contribute to F^−^ contamination in groundwater. However, the higher concentrations of F^−^ in groundwater is predominantly from geogenic sources and their exposure is a threat to human health^36^. The geogenic sources include presence of fluoride-bearing minerals, such as fluorite, amphiboles, topaz, apatite, fluorapatite, etc. in rocks, sediments and soils, evapotranspiration and atmospheric deposition^37–39^. Prominent anthropogenic sources are the applications of pesticides and phosphatic fertilizers, industrial effluents and landfills^40–42^.

Lower F^−^ concentrations (< 0.5 mg/L) in drinking water cause dental carries and concentrations between 0.6 and 1.5 mg/L are essential for bone formation and development of skeleton and teeth in the human body^4,24,43^. The long-term exposure to F^−^ concentrations above the recommended guideline/permissible limit (1.5 mg/L) may cause dental fluorosis, discoloration, pitting and mottling of teeth, skeleton fluorosis (4–8 mg/L), osteoporosis, arthritis, thyroid, rheumatic pain, kidney problem, muscle stiffness and abnormalities in red blood cells (> 10 mg/L)^38,44–47^. Globally, at least 200 million individuals are affected by acute fluorosis in 28 different nations due to the consumption of F^−^ contaminated groundwater^48^. In India alone, ~ 25 million individuals are affected by endemic fluorosis in 20 states besides ~ 66 million people in the risk of developing fluorosis, including ~ 6 million children below 14 years of age^49–52^. Mukherjee and Singh have made a detailed review of F^−^ contamination in groundwater in different states of India^53^.

**Figure 1:**
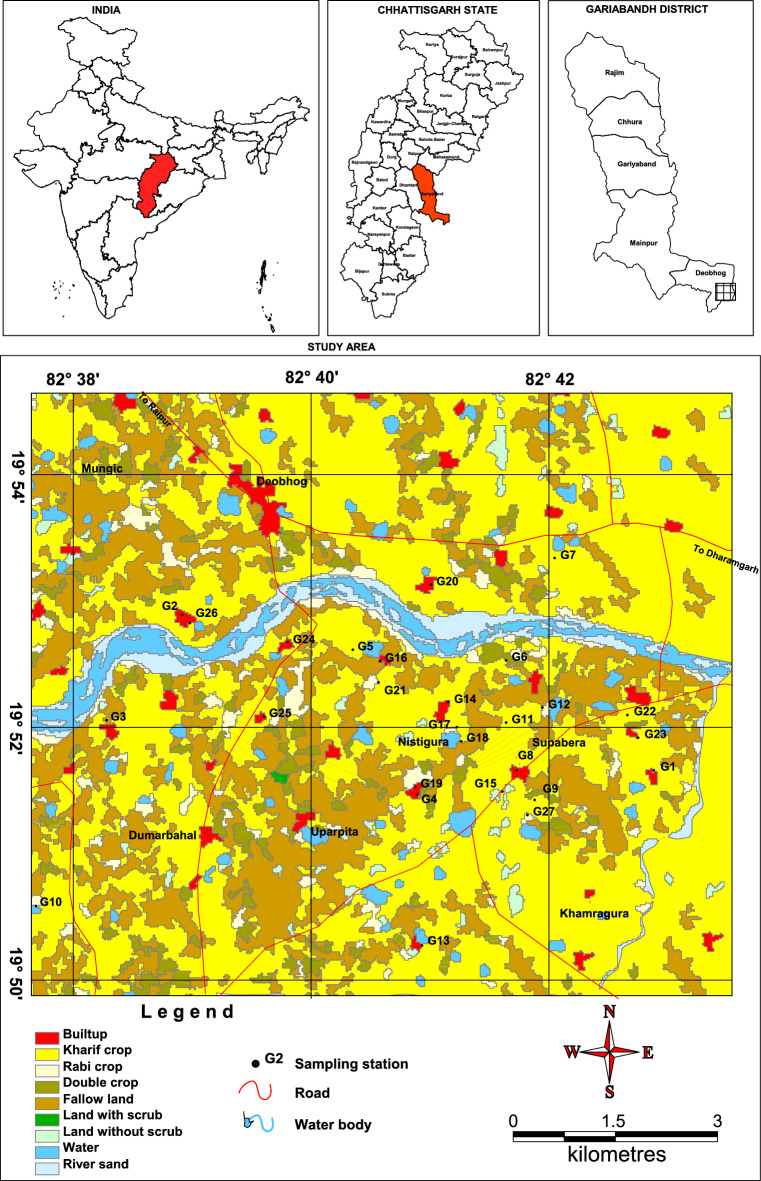
Groundwater sampling around village Supebeda in Chhattisgarh State, India: The village borders the state of Odisha on its east. Groundwater samples were collected from 27 locations marked by black dots. The map was prepared based on MapInfo 8.5 (https://www.precisely.com/product/precisely-mapinfo/mapinfo-pro).

Supebeda, the study area of this contribution, is a small village situated in the border of Chhattisgarh and Odisha States in a tribal stretch in central India (Fig. 1). Groundwater is the primary source of water in this region. In recent years, the local inhabitants have been facing numerous medical problems related to severe renal issues, kidney diseases and fluorosis. As per the media reports, there have been more than 100 causalities till date due to these diseases in recent years and around 300 villagers are battling for life. Thus, the study area has become a hotspot to understand the real reason for the peoples’ health problems. Several research organizations, such as the Indian Council of Medical Research, Geological Survey of India, Indira Gandhi Agricultural University, Chhattisgarh State Public Health and Engineering Department, Pandit Ravishankar Shukla University, National Institute Technology (Raipur), have already visited the village for investigation purposes. The research angles by many of these organizations have been genetic genesis, food habits, consumption of spurious liquor and other medicinal causes^54–56^. Presently, there is no literature available on the geochemistry of groundwater quality and associated health hazard risks to the local population. Preliminary sampling suggested NO_3_^−^ and F^−^ contamination in groundwater^57^. The present investigation, therefore, is aimed at making a detailed appraisal of the groundwater quality, non-carcinogenic health risk assessment in humans based on deterministic and probabilistic approaches, hydrochemical characterization, source apportionment of NO_3_^−^ and F^−^ through chemometric techniques and their relationship with other ions in groundwater.

## Materials and methods

### Study area

The study area, village Supebeda, lies between North latitudes 19° 50′ and 19° 54′ and East longitudes 82° 38′ and 82° 42′ occupying a geographical area of 3 km^2^ in the administrative block of Deobhog in Gariyaband district of Chhattisgarh State, India (Fig. 1). Situated on the bank of the river Tel, it borders the State of Odisha in the east. With a population of about 1200 people, it has nearly equal male–female sex ratio and literacy rate of 50.51%. The village has a Gram Panchayat (village council). The region is endowed with a sub-tropical monsoon climate with three distinct seasons: the southwest monsoon starts from mid-June to September; the winter season spreads from October to February and the summer season extends from March to mid-June. The average annual rainfall is 1200 mm, and the temperature in winter varies from 5 to 25 °C and in summer from 29 °C to 46 °C.

### Local geology

**Figure 2:**
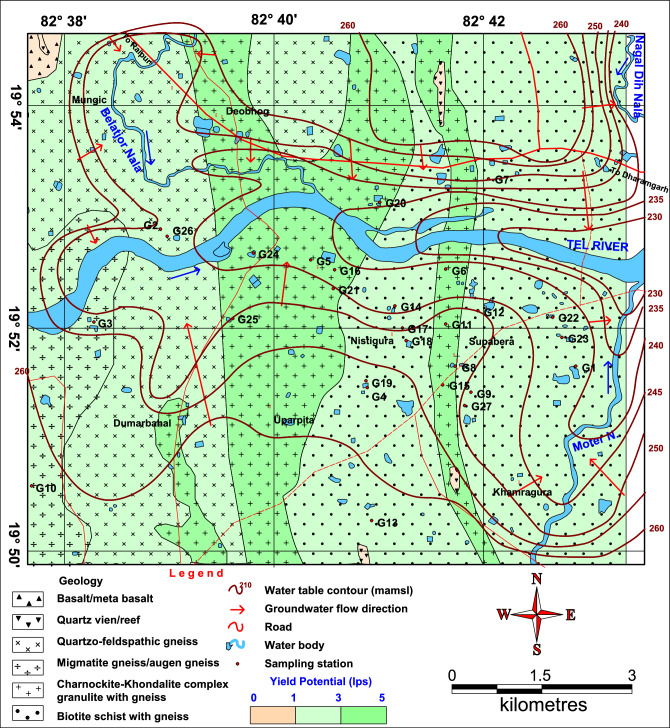
Geology and hydrogeology of the area around village Supebeda in Chhattisgarh State, India: The area represents a metamorphic terrain with a complex geology^58^. The arrow marks show the groundwater flow in different directions. Well drilled in the charnokite-khondalite complex are high-yielding with a yield potential of 3–5 L per second. The map was prepared based on MapInfo 8.5 (https://www.precisely.com/product/precisely-mapinfo/mapinfo-pro).

Gupta et al.^58^ and Neogi and Das^59^ have conducted detailed study on the geology of the area. As per this study, there are three major lithological units in the area from east to west, i.e., (i) migmatiticquartzo feldspathic gneiss, (ii) banded augen gneiss and (iii) hornblende granite (Fig. 2). Migmatiticquartzo feldspathic gneisses are grey-colored, medium-grained rocks with finely laminated alternations of felsic (quartz + plagioclase + K-feldspar: Qtz + Pl + Kfs) and mafic (Bt + Hbl-rich) bands. Leucocratic segregations are found extensively and are generally stromatically folded into or parallel to the layering. There is occasional presence of orthopyroxenes in migmatite gneisses as greasy, green patches with diffuse margins (‘patchy charnockite’). Bands of migmatized mafic granulites, metapelitic rocks (infrequently sapphirine-bearing) and rare calcsilicate granulites, besides isolated appearance blastoporphyritic charnockite, occur congruently with the gneisses.

Banded augen gneisses are pink-colored, medium- to coarse-grained rocks. The bandings within them are defined by mafic and felsic layers with K-feldspar (Kfs) augen and quartz lenticles. There is occasional occurrence of leucosomes in a narrow zone closer to the migmatitic quartzofeldspathic gneiss unit with sharp abetment to the west. The gneissic fabric generally precedes the leucosomes in banded augen gneiss. Hbl-rich and Pl + Cpx-rich layers are hosted thinly within banded gneisses. Amphibolites (Hbl + Pl ± Grt ± Cpx) and calc-silicate gneisses with these thin layers are mesoscopic to the regional scale bands.

Intruding into the banded gneiss is the pink-colored, coarse-grained hornblende granite that consists of microcline, quartz, hornblende, and biotite. With intense shearing and mylonitization along its eastern fringe, it has poor presence further westward.

### Hydrogeology

Groundwater occurs under unconfined condition in weathered portions of rocks and semi-confined to confined conditions in their fractured parts, i.e., in charnockite and khondalite, at depth. The shallow aquifer occurs within an average depth of 16 m. The configuration of water table in the shallow aquifer follows the topography due to which the groundwater movement is generally toward valleys or topographic lows. The water bodies, such as tanks, canals, and streams. also influence the occurrence and movement of groundwater in shallow aquifer. This aquifer is developed mostly by dugwells in the area with their depth ranging between 7 and 16 m. In general, the yield of dugwells ranges from 25 to 40 m^3^/day. Deeper aquifer in the area is formed mainly of granitic rocks and is developed by borewells with a depth range of 50–80 m. In general, the yield of borewells ranges from 85 to 430 m^3^/day.

The groundwater flow is analyzed based on the water table elevation contours. In northern part of the study area, groundwater flow is toward the south, i.e., the Tel River, while the flow is toward the north in the southern part. The water table elevations in the study area range between 240 and 260 m above mean sea level with northern part having higher groundwater table elevation. Transmissivity ranges from 15 to 45 m^2^/day in charnockite and khondalite and at favourable places it goes up to 100 m^2^/day. The potential fractures for boreholes up to 80 m depth are recorded at various depths, i.e., 40–45, 60–65, 75–80 m, and are 3–4 in numbers. Hydrogeology of the study area is shown in Fig. 2.

### Water sampling and analysis

Groundwater samples from twenty-seven locations were collected from the dugwells and borewells in and around the Supebeda area during pre-monsoon season (May 2020) (Fig. 1). Plastic bottles (HDPE) of 1000 ml capacity were used. These bottles were prewashed with HNO_3_ (10%) and rinsed with double deionised water. At the time of sample collection, groundwater sources were flushed for 10–15 min to obtain a fresh solution by removing the stagnant water in the pipe. The sampling bottles were thoroughly rinsed 2–3 times with the fresh groundwater to be collected to preserve the original characteristics of the sampled water. Some basic parameters, such as pH, electrical conductivity (EC) and total dissolved solids (TDS), were immediately measured onsite after the collection of groundwater samples using a pH/EC/TDS meter (Hanna HI 9811-5). Whatman filter paper (0.45 μm) was used to remove the suspended particulate matter. The samples were preserved by acidifying (pH ~ 2 with HNO_3_) and kept at a temperature of 4 °C. Standard protocol prescribed by the American Public Health Association was followed for the investigation of major cations (Ca^2+^, Mg^2+^, Na^+^, and K^+^) and anions (HCO_3_^−^, Cl^−^, SO_4_^2−^, F^−^, and NO_3_^−^). Merck-GR grade chemicals and reagents were used to prepare the chemical solutions using double deionized water. All the glassware and apparatus were soaked with 10% hydrochloric acid (HCl) for one day and cleaned with double deionized water. Blank samples were prepared from the stock solutions of each parameter for instrumental calibration. The accuracy of analysing datasets was computed using the charge balance error (CBE) equation (Eq. 1), and each sample value was within its error limit of ± 5%^60^.

1 $${\text{CBE}}\% = \frac{{\sum \left( {Cations} \right)meq/L - \sum \left( {Anions} \right)meq/L}}{{\sum \left( {Cations} \right)meq/L + \sum \left( {Anions} \right)meq/L}} \times 100$$

### Human health risk assessment (HHRA)

Human health risk assessment (HHRA) is the quantitative risk analysis of potentially harmful chemical parameters present in water on human health through various pathways and specific time periods^61,62^. It has four distinct steps: (1) hazard identification, (2) exposure assessment, (3) dose–response assessment and (4) risk characterization^4,63^.

The significant pathways for risk analysis on human health from chemical exposure are ingestion and dermal contact. In the present study, the average daily dose (*ADD*) of ingestion and dermal pathways for target chemicals, namely NO_3_^−^ and F^−^, are employed to determine the non-carcinogenic HHRA as shown in Eqs. (2) and (3)^64^. The assessments of *ADD*_*ingestion*_ and *ADD*_*dermal*_ are computed on four different age groups, i.e., infants (< 1 year), children (1–11 years), teens (11–18 years) and adults (above 18 years). The adverse impact of the target parameters on human health may vary due to physiological and behavioural attributes, organ development factors and tolerance responses to the specific chemicals in the human body.

2 $$ADD_{ingestion} = \frac{{C_{M} \times IR_{w} \times EF_{r} \times ED}}{{BW \times AT_{r} }}$$

3 $$ADD_{dermal} = \frac{{C_{M} \times SA \times K_{p} \times EF_{r} \times ED \times ET \times CF}}{{BW \times AT_{r} }}$$

(The parameters/variables used in these equations are defined in Supplementary Table S1).

The ratio of the potential adverse non-carcinogenic risk from each exposure pathway (ingestion and dermal) with respect to the corresponding reference dose of a chemical parameter is estimated through hazard quotient (*HQ*)^64^, as shown in Eqs. (4)–(5). Hazard index (*HI*) is the combined non-carcinogenic hazard risks of a particular parameter from all different possible exposure routes^65,66^. Both *HI* and *HQ* are unitless values. When *HQ* > 1, it is indicative of potential health effects from a specific exposure route^67^. Similarly, the value of *HI* > 1 depicts the adverse non-carcinogenic toxicity in each target age group^67^.

4 $$HQ_{ingestion } = \frac{{ADD_{ingestion} }}{{RfD_{i} }}$$

5 $$HQ_{dermal } = \frac{{ADD_{dermal} }}{{RfD_{d} }}$$

6 $$HI_{M } = \mathop \sum \limits_{i = 1}^{n} HQ_{ingestion} + \mathop \sum \limits_{i = 1}^{n} HQ_{dermal}$$

where $HI_{M }$ indicates the total hazard index of a specific parameter, and *i* represents the exposure route of a specific subpopulation group considered in the present study (Eq. 6). The ingestion and dermal pathways reference dose (*RfD*) values for NO_3_^−^ and F^−^ are 1.6 mg/kg per day and 0.06 mg/kg per day, respectively^4,68–70^.

Both deterministic and probabilistic approaches are applied to determine the potential non-carcinogenic HHRA in the present study. The deterministic approach simply incorporates fixed values on the mathematical formula developed by USEPA for different exposure pathways^71^. The point estimation results generate only a single value that may underestimate or overestimate the risk analysis. Normally, the values of the variables of the point estimation vary with respect to climatic conditions, place, time, chemical concentrations in water and receptor types (i.e., body weight, exposure frequency and different subpopulation groups)^72^, but since the uncertainty of the deterministic model considers only a fixed value for every input variable, this technique is a conservative risk assessment approach.

Probabilistic technique, namely Monte Carlo Simulation (MCS), is an alternative statistical model that offers a sound methodology and provides holistic information for risk assessment suggested by USEPA^72^. Monte Carlo Simulation is a computer software application configuring a statistical distribution array in the form of probabilistic approximation of a mathematical equation to generate more corroborated reproducibility results and reduces the uncertainty associated in risk analysis^4^. Oracle Crystal Ball software version (11.1.2.4.850) is used for the MCS study. The operation of MCS requires prearrangement of input variables/parameters with respect to their maximum, minimum, mean, and standard deviation (SD) values to define best-fitted statistical distribution types to generate their probability distribution functions (PDFs)^72^. The input parameters, such as ingestion rate (*IR*_*w*_), exposure frequency (*EF*), exposure duration *(ED)*, expose skin surface area (*SA*), exposure time (*ET*) and body weight (*BW*), generally have 10,000 repetitions for the computation of risks from oral ingestion and dermal contact for each subpopulation group. Thus, the numerical stability of MCS is obtained at 10,000 permutations for *HQ* and *HI*^4,73,74^. The sensitivity analysis is also employed to extract the significant input variables impacting the outcome of a simulation model for potential risks.

In this work, the target parameters, i.e., NO_3_^−^ and F^−^, are defined by the auto-select to determine the best-fitted probability distribution pattern based on their concentration values. Their goodness of fit (GoF) statistical outcomes are presented in Table 1. The values and types of distribution of various input variables for ingestion and dermal pathways for the deterministic and probabilistic models are provided in Supplementary Table S1.

**Table 1:** Best fitted and goodness of fit (GoF) outcomes of the probability distribution of Nitrate and Fluoride in the groundwater around village Supebeda in Chhattisgarh State, India.

Parameters	Distribution types and their parameter values	Anderson–Darling test	Anderson–Darling test (*p* value)	Kolmogorov–Smirnov test	Kolmogorov–Smirnov test (*p* value)	Chi-square test	Chi-square test (*p* value)
Premonsoon							
Nitrate	Logistic (Mean = 34.25, Scale = 24.04)	14,593	0.000	0.1940	0.000	14.667	0.001
Fluoride	Uniform (Min = 0.01, Max = 1.97)	0.5670	0.543	0.1815	0.250	5.0370	0.081

### Chemometric analysis

Chemometric statistical models, such as principal component analysis (PCA) and cluster analysis (CA), are widely used by many researchers to distinguish among the probable sources of chemical parameters in water^11,75–78^. Principal component analysis enables extraction of valuable information and better interpretation of statistically significant parameters from large, complex datasets^79^. The present study uses z-scale standardization of all chemical parameters to generate dimensionless values^80–82^. Varimax rotation method has been employed to extract the principal components (PCs). The PCs with eigenvalues > 1 are statistically significant for interpreting the hidden factors in water quality^83,84^.

Cluster analysis has been used to create similar groups from a different set of objects or variables^85^. Ward’s linkage and squared Euclidean distance have been applied on z-transformation data to obtain different clusters^86^. The cluster significance has been assessed using Sneath’s test method^87^. Minitab 17 and MS Office 2021 have been employed to perform the statistical analysis.

## Results and discussions

Table S2 lists the concentrations of various physicochemical parameters in analyzed groundwater samples. Table 2 gives the statistical description of physicochemical parameters [range, mean, and standard deviation (SD)] and percentage of samples above the BIS^46^ and WHO^45^ standards. Water samples are neutral to slightly alkaline in nature with the pH values ranging from 7.2 to 8.3 with a mean of 7.9 (mean ± SD = 7.9 ± 0.3). EC values show wide variation from 313.0 to 3446.0 µS/cm with 11.11% samples above the guideline value of 1500 µS/cm^45^. High EC values at some locations cause salinity due to excessive mineralization in groundwater. The water quality classification based on EC values^88^ indicates that 62.96% of the samples are moderately saline, 26.63% are medium to highly saline and 7.41% are highly saline for irrigation purposes (Table S3). Further, classification by FAO^89^ shows that 7.41% samples are above the standard EC range (0–3000µS/cm) for irrigational use (Table 2).

**Table 2:** Descriptive statistics of chemical parameters of groundwater samples collected from Supebeda, district Gariyaband, Chhattisgarh, India.

Parameter	BIS^46^ standards		FAO^89^	Premonsoon				
			Standards (usual range for irrigation use)	Range	Mean ± SD	% of sample above BIS^46^ and WHO^45^ Standards		% of sample above FAO^89^ Standards
	AL	PL				AL	PL	
Physical parameters								
pH	6.5–8.5		6.5–8.4	7.2–8.3	7.9 ± 0.3	NIL		NIL
EC	1500^a^		0–3000	313.0–3446.0	941 ± 795	11.11% (3)		7.4% (2)
TDS	500	2000	0–2000	200.32–2205.44	602.2 ± 509.0	22.22% (6)	11.11% (3)	11.11% (3)
TH	200	600	–	65.0–755.0	257 ± 178	33.33% (9)	11.11% (3)	–
Major cations								
Ca^2+^	75	200	0–400	20.0–214.0	67 ± 53	29.6% (8)	3.7% (1)	NIL
Mg^2+^	30	100	0–60	3.6–52.8	21 ± 13.2	11.11% (3)	NIL	NIL
Na^+^	200^a^		0–920	16.4–185.5	65 ± 43.9	NIL		NIL
K^+^	12^a^		–	0.6–11.4	2.2 ± 2.1	NIL		–
Major anions								
HCO_3_^−^	500^a^		0–610	85.0–519.0	297 ± 109	3.7% (1)		NIL
Cl^−^	250	1000	0–1063	7.1–408.3	73.8 ± 109.2	11.11% (3)	NIL	NIL
SO_4_^2−^	200	400	0–960	4.8–105.5	29 ± 30.6	NIL	NIL	NIL
NO_3_^−^	45		0–45	0–128.3	39 ± 40	37.0% (10)		37.0% (10)
F^−^	1	1.5	0–20	0–1.9	0.9 ± 0.6	14.8% (4)	25.9% (7)	NIL

AL and PL stands for acceptable limits and permissible limits in the absence of alternative source of water (BIS^46^).

^a^Indicates parameters guideline values as per WHO^45^.

Groundwater samples with TDS values above acceptable limit of 500 mg/L and permissible limit of 2000 mg/L^46^ for drinking purposes are 22.22% and 11.11%, respectively, of all collected samples. These 11.11% of the samples are above the normal range of 0–2000 mg/L^89^ for irrigational use as well. As per Davis and DeWiest^90^, about 63% of the samples are within the desirable limit for drinking purposes and about 26% in between desirable and permissible limit of 1000 mg/L (Table S3). Further, the TDS classification by Freeze and Cherry^91^ indicates that majority of the groundwater samples (88.89%) falls under freshwater and the rest 11.11% under brackish water category (Table S3).

Total hardness (TH) values vary from 65.0 to 755.0 mg/L with 33.33% and 11.11% samples above the acceptable (200 mg/L) and permissible limits (600 mg/L), respectively^46^. The elevated level of TH is primarily linked with the excess concentrations of Ca^2+^, Mg^2+^ and HCO_3_^−^ ions in groundwater^11,76^. Classification of groundwater based on TH values by Sawyer and McCarty^92^ divulges that 3.70% of the samples are soft, 22.22% are moderately hard, 44.44% are hard and 29.63% are very hard in nature (Table S3). Further, TDS versus TH plot depicts that the groundwater is fresh to brackish water types with moderately hard to very hard in nature (Fig. S1). Sindhu concludes that the prolonged consumption of very hard water is associated with calcification of arteries, urolithiasis, anencephaly, and gastrointestinal tract irritation^93^. Box-Whisker plot shows the relative abundance and dominance of various cations (Ca^2+^ > Na^+^ > Mg^2+^ > K^+^) and anions (HCO_3_^−^ > Cl^−^ > NO_3_^−^ > SO_4_^2−^ > F^−^) in groundwater (Fig. S2).

### Major parameters

About 26.9% and 3.7% samples show Ca^2+^ contents above the acceptable limit of 75 mg/L and permissible limit of 200 mg/L, respectively^46^. The Mg^2+^ mean ± SD is 21 ± 13.2 with 11.11% of samples above the acceptable limit of 30 mg/L^46^ (Table 2). The alkali metals, i.e., Na^+^ and K^+^, are within their respective guideline values (200 mg/L and 12 mg/L)^45^. HCO_3_^−^ concentrations range from 85 to 519 mg/L with 3.7% of samples above the guideline value of 500 mg/L^45^. Chloride (Cl^−^) concentrations vary from 7.1 to 408.3 mg/L, with 11.11% of samples above the acceptable limit of 250 mg/L^46^. The excess concentrations of Ca^2+^, Mg^2+^, HCO_3_^−^ and Cl^−^ ions are the key chemicals resulting hardness of groundwater^24^. The level of SO_4_^2−^ ions in groundwater is within the acceptable limit of 200 mg/L^46^. The concentrations of cations (Ca^2+^, Mg^2+^, and Na^+^) and anions (HCO_3_^−^, Cl^−^, and SO_4_^2−^) are within their normal ranges for irrigational use (Table 2)^89^.

### Health risk parameters

Consumption of NO_3_^−^ and F^−^-rich water causes various health risks in humans. The NO_3_^−^ content in groundwater in Supebeda exceeds its guideline value of 45 mg/L for drinking and irrigation purposes^46,89^ in 37.0% of samples (Table 2). The classification of NO_3_^−^ concentrations based on Adimalla^43^ signifies that 62.96% of the samples have no risk (< 45 mg/L), 29.63% of samples have high risk (45–100 mg/L) and the remaining 7.41% of samples have very high risk to human health (> 100 mg/L) (Table S4).

The range of F^−^ concentrations varies from 0 to 1.9 mg/L with 14.8% and 25.9% of samples above the acceptable limit of 1.0 mg/L and permissible limit of 1.5 mg/L, respectively^46^. Although about 55.56% of samples have the required F^−^ level (0.6–1.5 mg/L) for human health, as per Adimalla^43^, 22.22% of samples may cause dental caries (< 0.5 mg/L) and an equal percent dental fluorosis (1.6–2.0 mg/L) (Table S4).

### Health risk assessment (HRA)

Table ﻿S5 provides the calculated average daily dose (ADD) values of NO_3_^−^ and F^−^ through ingestion and dermal contact of groundwater using deterministic and probabilistic approaches for different age groups**.** Tables 3 and 4 show estimates of the non-carcinogenic HRA with respect to hazard quotient ($HQ$) and hazard index ($HI$) parameters, respectively. The deterministically calculated mean, median, 5th percentile (minimum) and 95th percentile (maximum) values of $HQ_{ingestion}$, $HQ_{dermal}$ and $HI$ for NO_3_^−^ and F^−^ are relatively more than those of the probabilistically estimated values in all target population groups. The mean and 95th percentile of $HQ_{{{\text{NO}}_{3}^{ - } }}$ for ingestion pathway are above the acceptable limit (i.e., $HQ$ > 1) in the deterministic study, which indicates that the potential non-carcinogenic risk shall affect the larger sections in all target populations. On the other hand, the probabilistically calculated $HQ_{{{\text{NO}}_{3}^{ - } }}$ for ingestion pathway is above the threshold limit (i.e., $HQ$ > 1) only at 95th percentile, which shows that the clinical risk of non-carcinogenic effect is a concern to the sensitive sections of people in all subpopulation groups at the extreme point (Table 3).

**Table 3:** Statistical description of deterministically and probabilistically calculated hazard quotient (*HQ*) for ingestion and dermal pathways in different age groups.

Premonsoon	Parameter	Deterministic value (ingestion)					Risk certainty level (RCL) %	Probabilistic value (ingestion)					Risk certainty level (RCL) %
Age group		Mean	Median	SD	5th percentile	95th percentile	*HQ* > 1	Mean	Median	SD	5th percentile	95th percentile	*HQ* > 1
Infants	$HQ_{{{\text{NO}}_{3}^{ - } }}$	2.20E+00	1.22E+00	2.26E+00	1.69E−02	5.88E+00	51.85%	9.04E−01	5.27E−01	2.49E+00	− 7.45E−01	3.74E+00	34.02%
Children		1.43E+00	7.90E−01	1.46E+00	1.10E−02	3.81E+00	48.15%	5.47E−01	3.52E−01	1.45E+00	− 5.54E−01	2.24E+00	23.00%
Teens		1.03E+00	5.70E−01	1.06E+00	7.91E−03	2.75E+00	37.04%	3.84E−01	2.51E−01	1.01E+01	− 3.92E−01	1.54E+00	13.16%
Adults		1.11E+00	6.13E−01	1.14E+00	8.52E−03	2.96E+00	40.74%	3.25E−01	2.43E−01	9.26E−01	− 3.72E−01	1.45E+00	11.62%
Infants	$HQ_{{F^{ - } }}$	1.41E+00	1.05E+00	8.84E−01	3.01E−01	2.82E+00	66.67%	7.32E−01	4.92E−01	1.54E+00	4.16E−02	2.23E+00	24.17%
Children		9.14E−01	6.83E−01	5.73E−01	1.95E−01	1.82E+00	33.33%	4.68E−01	3.53E−01	8.95E−01	3.12E−02	1.30E+00	10.55%
Teens		6.59E−01	4.92E−01	4.13E−01	1.41E−01	1.31E+00	25.93%	2.86E−01	2.03E−01	5.53E−01	9.03E−03	8.36E−01	2.00%
Adults		7.09E−01	5.30E−01	4.44E−01	1.51E−01	1.42E+00	33.33%	2.76E−01	2.02E−01	5.27E−01	9.08E−03	7.85E−01	1.25%

	Parameter	Deterministic value (dermal)					*HQ* > 1	Probabilistic value (dermal)					*HQ* > 1
Infants	$HQ_{{NO_{3}^{ - } }}$	6.65E−03	3.68E−03	6.81E−03	5.11E−05	1.77E−02	NIL	4.09E−04	2.63E−04	1.06E−03	− 3.64E−04	1.63E−03	NIL
Children		4.99E−03	2.76E−03	5.11E−03	3.83E−05	1.33E−02	NIL	4.54E−04	2.94E−04	1.19E−03	− 3.96E−04	1.77E−03	NIL
Teens		4.73E−03	2.62E−03	4.85E−03	3.64E−05	1.26E−02	NIL	3.54E−04	2.38E−04	9.20E−04	− 3.22E−04	1.40E−03	NIL
Adults		5.71E−03	3.16E−03	5.85E−03	4.39E−05	1.52E−02	NIL	3.16E−04	2.13E−04	8.22E−04	− 3.21E−04	1.26E−03	NIL
Infants	$HQ_{{F^{ - } }}$	4.25E−03	3.18E−03	2.66E−03	9.08E−04	8.89E−03	NIL	3.30E−04	2.46E−04	6.34E−04	2.38E−05	9.15E−04	NIL
Children		3.19E−03	2.38E−03	2.00E−03	6.81E−04	6.37E−03	NIL	3.63E−04	2.66E−04	7.04E−04	2.42E−05	1.02E−03	NIL
Teens		3.03E−03	2.26E−03	1.90E−03	6.46E−04	6.04E−03	NIL	2.64E−04	1.94E−04	5.03E−04	9.71E−06	7.51E−04	NIL
Adults		3.65E−03	2.73E−03	2.29E−03	7.80E−04	7.29E−03	NIL	2.41E−04	1.79E−04	4.62E−04	7.47E−06	6.87E−04	NIL

**Table 4:** Statistical description of deterministically and probabilistically calculated hazard index (*HI*) for ingestion and dermal pathways in different age groups.

Age group	Pathways (ingestion + dermal)	Deterministic value					Risk certainty level (RCL) (%)	Probabilistic value					Risk certainty level (RCL) (%)
		Mean	Median	SD	5th percentile	95th percentile	*HI* > 1	Mean	Median	SD	5th percentile	95th percentile	*HI* > 1
Infants	$HI_{{{\text{NO}}_{3}^{ - } }}$	2.21E+00	1.22E+00	2.27E+00	1.70E−02	5.90E+00	51.85%	9.05E−01	5.27E−01	2.49E+00	− 7.45E−01	3.74E+00	34.03
Children		1.43E+00	7.93E+00	1.47E+00	1.10E−02	3.83E+00	48.15%	5.48E−01	3.35E−01	1.45E+00	− 5.55E−01	2.24E+00	23.01
Teens		1.03E+00	5.72E−01	1.06E+00	7.95E−03	2.76E+00	40.74%	3.84E−01	2.51E−01	1.02E+00	− 3.92E01	1.54E+00	13.17
Adults		1.11E+00	6.16E−01	1.14E+00	8.56E−03	2.97E+00	40.74%	3.52E−01	2.43E−01	9.26E−01	− 3.72E−01	1.45E+00	11.62
Infants	$HI_{{{\text{F}}^{ - } }}$	1.41E+00	1.06E+00	8.87E−01	3.02E−01	2.82E+00	66.67%	7.32E−01	4.92E−01	1.54E+00	4.18E−02	2.23E+00	24.17
Children		9.17E−01	6.85E−01	5.75E−01	1.96E−01	1.83E+00	37.04%	4.68E−01	3.53E−01	8.95E−01	3.15E−02	1.30E+00	10.56
Teens		6.62E−01	4.94E−01	4.15E−01	1.41E−01	1.32E+00	25.23%	2.86E−01	2,04E−01	5.53E−01	9.34E−03	8.36E−01	2.00
Adults		7.13E−01	5.33E−01	4.47E−01	1.52E−01	1.42E+00	33.33%	2.76E−01	2.02E−01	5.27E−01	9.34E−03	7.85E−01	1.25

In deterministic estimate, the ingestion route of $HQ_{{{\text{F}}^{ - } }}$ shows mean and 95th percentile above the safety limit ($HQ$ > 1) only in infants, and rest of the population groups (Children, Teens, and Adults) have $HQ_{{{\text{F}}^{ - } }}$ > 1 in 95th percentile. In probabilistic study, the threat of non-carcinogenic hazard divulges at the maximum point ($HQ_{{{\text{F}}^{ - } }}$ 95th percentile > 1) through ingestion pathway in the infants and children's groups (Table 3). In dermal contact, the deterministically and probabilistically calculated mean, median, 5th percentile and 95th percentile values $HQ_{{{\text{NO}}_{3}^{ - } }}$ and $HQ_{{{\text{F}}^{ - } }}$ are less than the threshold limit ($HQ$ < 1) in all target population groups. This indicates that there is no potential non-carcinogenic health risk through dermal contact from the indicator parameters (Table 3).

Risk certainty level (*RCL*) is assessed to generate the likelihood percentage scenarios of non-cancer hazard quotient risk above the threshold value ($HQ$ > 1) in all individual datasets of a particular pathway. It is always advantageous to determine the *RCL* value in HRA for any exposure pathway, even if the mean, 5th percentile and 95th percentile values of different age groups are below their threshold limits. Among the target age groups, the order of deterministic *RCL* ($HQ$ > 1) for NO_3_^−^ and F^−^ through the ingestion route is infants ($HQ_{{{\text{NO}}_{3}^{ - } }} =$ 51.85% and $HQ_{{{\text{F}}^{ - } }}$ = 66.67%) > children ($HQ_{{{\text{NO}}_{3}^{ - } }}$ 48.15% and $HQ_{{{\text{F}}^{ - } }}$ = 33.33%) > adults ($HQ_{{{\text{NO}}_{3}^{ - } }} =$ 40.74% and $HQ_{{{\text{F}}^{ - } }}$ = 33.33%) > teens ($HQ_{{{\text{NO}}_{3}^{ - } }}$ = 37.04% and $HQ_{{{\text{F}}^{ - } }}$ = 25.93%) (Table 3). Similar findings of NO_3_^−^ and F^−^ non-carcinogenic health risk for groundwater ingestion pathways are found in Jiangcungou, Northwest China (i.e., children > adults > teenagers)^1^ and Nalagarh valley, Himachal Pradesh, India (i.e., infants > children > adults > teenagers)^4^. On the other hand, the probabilistic *RCL* ($HQ$ > 1) orders for NO_3_^−^ and F^−^ through ingestion pathway are infants ($HQ_{{{\text{NO}}_{3}^{ - } }}$ = 34.02% and $HQ_{{{\text{F}}^{ - } }}$ = 24.17%) > children ($HQ_{{{\text{NO}}_{3}^{ - } }}$ = 23.00% and $HQ_{{{\text{F}}^{ - } }}$ = 10.55%) > teens ($HQ_{{{\text{NO}}_{3}^{ - } }}$ = 13.16% and $HQ_{{{\text{F}}^{ - } }}$ = 2.00%) > adults ($HQ_{{{\text{NO}}_{3}^{ - } }}$ = 11.62% and $HQ_{{{\text{F}}^{ - } }}$ = 1.25%) (Table 3).

The deterministic and probabilistic *RCLs* ($HQ$ > 1) indicate trivial non-carcinogenic risks from the indicator parameters (NO_3_^−^ and F^−^) through the dermal route. Therefore, the perusal of Table 3 shows that NO_3_^−^ and F^−^ exposure through direct groundwater consumption has higher non-carcinogenic $HQ$ by several orders of magnitude than that of the dermal route in all age groups. Liu get similar findings of non-cancerous health risks from the groundwater of Weining plain, China^72^. Further, among the indicator parameters, the mean, median and 95^th^ percentile values of $HQ_{{{\text{NO}}_{3}^{ - } }}$ are more than those of $HQ_{{{\text{F}}^{ - } }}$ through the groundwater ingestion pathway within each stratified age group in both deterministic and probabilistic approaches (Table 3).

### Hazard index (HI)

The non-carcinogenic $HI$ is the combination of non-carcinogenic hazard quotient risk factors of each indicator parameter (NO_3_^−^ or F^−^) through multi-exposure pathways (ingestion and dermal) of groundwater, as shown in Table 4. The mean, median and 95th percentile values of infants and children in the deterministic result exceed the safety reference level of $HI_{{{\text{NO}}_{3}^{ - } }}$ > 1, divulging prominent threat level of non-carcinogenic HHR from NO_3_^−^ in these age groups. The remaining population groups (teens and adults) in deterministic study and all the target population groups in probabilistic estimate reveal the non-carcinogenic risk of NO_3_^−^ at 95th percentile values only ($HI_{{{\text{NO}}_{3}^{ - } }}$ > 1).

With respect to F^−^, in the deterministic study, the potential non-cancerous effect is prominent in infants since the mean, median and 95th percentile values are above the safe reference limit (i.e., $HI_{{{\text{F}}^{ - } }}$ > 1), but the rest of the subpopulation groups show $HI_{{{\text{F}}^{ - } }}$ > 1 in 95th percentile only, which shows that the threat of health risk is still persistent in the sensitive sections of the stratified age groups at the extreme value. On the other hand, in the probabilistic estimate, the $HI_{{{\text{F}}^{ - } }}$ results indicate that the infants and children’s groups are at the risk of non-carcinogenic effect at 95th percentile values, i.e., $HI_{{{\text{F}}^{ - } }}$ > 1.

Accordingly, in the deterministic output, the *RCL* magnitude of non-carcinogenic $HI_{{{\text{NO}}_{3}^{ - } }}$ risk stands at infants (51.85%) > children (48.15%) > teens (40.74%) = adults (40.74%), and that of $HI_{{{\text{F}}^{ - } }}$ at infants (66.67%) > children (37.04%) > adults (33.33%) > teens (25.23%) (Table 4). The probabilistically calculated *RCL* health risks in the subpopulation groups are in the following order: infants ($HI_{{{\text{NO}}_{3}^{ - } }}$ = 34.03% and $HI_{{{\text{F}}^{ - } }}$ = 24.17%) > children ($HI_{{{\text{NO}}_{3}^{ - } }}$ = 23.01% and $HI_{{{\text{F}}^{ - } }}$ = 10.56%) > teens ($HI_{{{\text{NO}}_{3}^{ - } }}$ = 13.17% and $HI_{{{\text{F}}^{ - } }}$ = 2.00%) > adults ($HI_{{{\text{NO}}_{3}^{ - } }}$ = 11.62% and $HI_{{{\text{F}}^{ - } }}$ = 1.25%).

The deterministic *RCL* for $HI$ is more than the probabilistic *RCL* in all age groups divulging that the deterministic estimation is based on the extreme (single point) values (please see Table S1, fifth column) for all input variables individually at different concentration levels of the indicator parameters. Since these extreme (single point) values may not always represent the actual field conditions, the deterministic estimates often lead to overestimation of the output results (Table 4). Therefore, the deterministic approach cannot cater to the holistic scenario of risk assessment for the inclusive members of the population interests due to differences in person-to-person characteristics and dynamism prevailing in the environment.

The probabilistic approach gives a range of values to choose from depending on the most likelihood field conditions (please see Table S1, eighth column). Therefore, the probability approximation of events reduces the uncertainties by providing more accurate and prospective risk assessment outcomes than those of the conventional deterministic approach. Liu^72^ too conclude that the health risk assessment in groundwater through probabilistic simulation provides more comprehensive results.

The present study, however, suggests that the HRA of the indicator parameters should be studied using both deterministic and probabilistic approaches mutually to obtain more holistic outputs, thereby reducing the uncertainties and overcoming the conservative risk analysis of the point estimation. In a similar line, Kaur^25^ conclude that the deterministic and probabilistic methods may be studied independently to assess non-carcinogenic HHRA (NO_3_^−^ and F^−^) in groundwater.

### Sensitivity and uncertainty analysis

**Figure 3:**
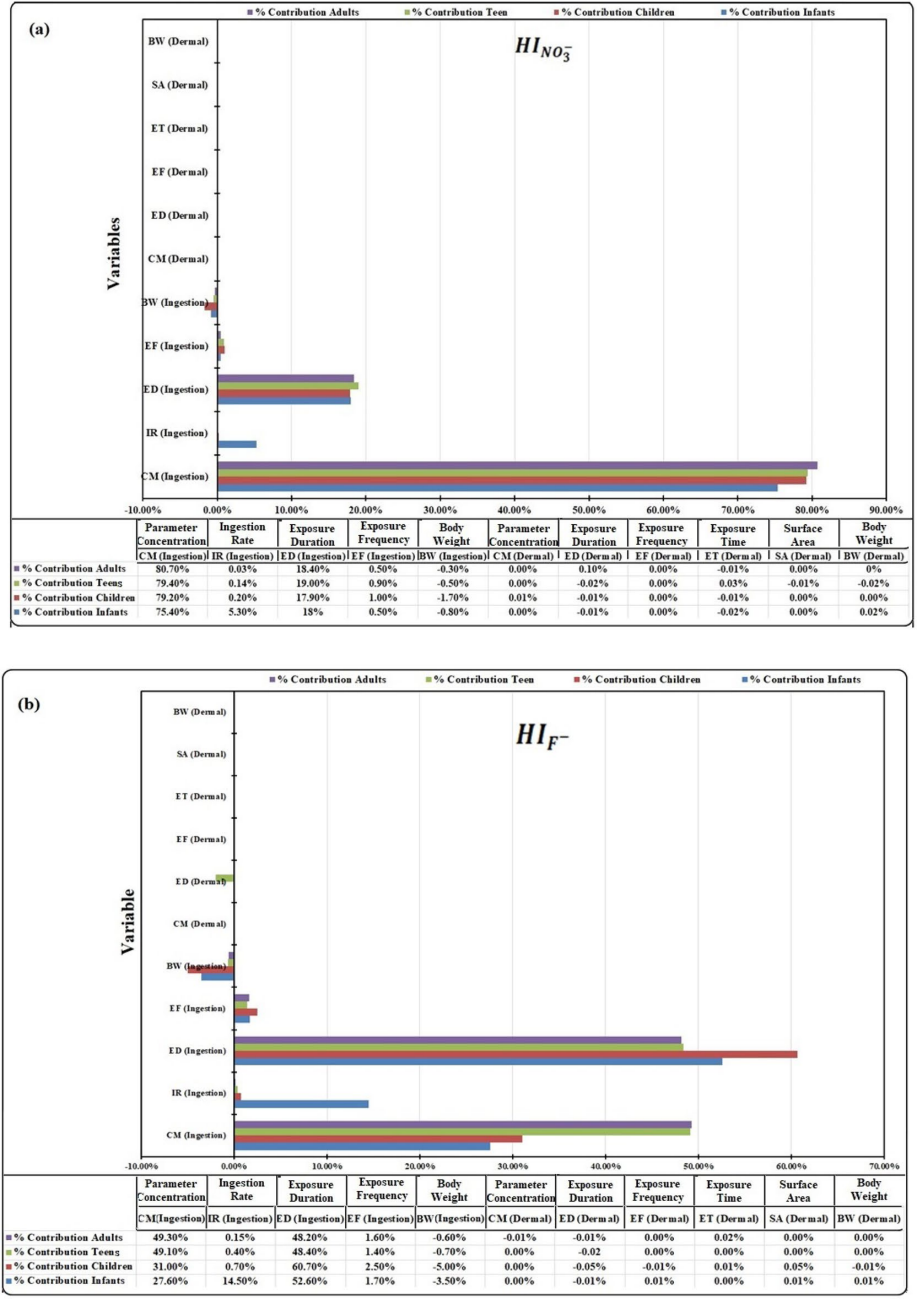
Tornado plots illustrating sensitivity analysis of input variables to the non-carcinogenic hazard index (*HI)* of groundwater: (**a**) NO_3_^−^ and F^−^ ingestion and (**b**) for dermal contact: This sensitivity analysis figure is drawn by the probabilistic approach using the Monte Carlo Simulation (MCS) technique to extract the most influential input variables for the non-carcinogenic risk prediction for human health. The length of horizontal bars indicates the percentage contribution of various input variables to extract the non-carcinogenic hazard index (*HI*) of different age groups.

Deterministic technique does not provide any provision for sensitivity and uncertainty analysis. Therefore, sensitivity analysis has been carried out in the probabilistic process of working using the Monte Carlo Simulation (MCS) approach to extract the most influential input variables for the non-carcinogenic risk prediction. Figure 3a,b represents the tornado plots showing the percentage scales of all input variables for non-carcinogenic $HI_{{{\text{NO}}_{3}^{ - } }}$ and $HI_{{{\text{F}}^{ - } }}$ in the stratified age groups. The sensitivity analysis validates that the variables of dermal route are not vividly influenced in the overall contribution of non-carcinogenic $HI$ in all subpopulation groups and that the input variables of the ingestion pathway have more potential non-carcinogenic health effects than those of the dermal contact. The $HQ$ results are further supported and validated by the sensitivity analysis of tornado plots. For $HI_{{{\text{NO}}_{3}^{ - } }}$ sensitivity output, the parameter concentration (C_M *ingestion*_) is the most influential variable followed by exposure duration (ED_*ingestion*_) with minor contributions from ingestion rate (IR_*ingestion*_) and exposure frequency (EF_*ingestion*_) in all target populations. It indicates that higher NO_3_^−^ content in ingested water will have more health implications, but as per Carlsson^94^, 60–70% of the intake NO_3_^−^ dose is generally excreted within the first 23 h in urine. Therefore, possibly the clinical NO_3_^−^ toxicity in humans is less significant because of the limited exposure duration of NO_3_^−^ intake dose in the body.

The results of sensitivity analysis $HI_{{{\text{F}}^{ - } }}$ for infants and children stand in the order of ED_*ingestion*_ > C_M*ingestion*_ > IR_*ingestion*_ > EF_*ingestion*_. In infants and children, 80% of the oral F^−^ intake is absorbed in the body with storage in the bones and^95^. Thus, exposure duration is the most significant input variable due to high retention of F^−^ intake dose in infants and children. The tornado $HI_{{{\text{F}}^{ - } }}$ plots for teens and adults show the percentage of contribution variables as C_M *ingestion*_ > ED_*ingestion*_ > IR_*ingestion*_ > EF_*ingestion*_. For teens and adults, ~ 50% of an orally ingested F^−^ is retained in the body^95,96^. Thus, the lower retention potential of F^−^ dose in teens and adults compared to that in infants and children indicates that the parameter concentration is the main driving force for fluoride toxicity in the sensitivity outputs. The body weight (BW_*ingestion*_) variable negatively infers non-carcinogenic $HI_{{{\text{NO}}_{3}^{ - } }}$ and $HI_{{{\text{F}}^{ - } }}$ simulations in all age groups (Fig. 3a,b).

Uncertainty analysis is crucial in determining the conservatism, ramification, and certainty accuracy level of the risk analysis results^97^. In this study, the application of MCS is notably enhanced to identify and quantify the uncertainties in the non-cancer HRA. Nevertheless, there are still other uncertainties that remain unaccounted in the model input variables, thereby limiting the validity of the whole scenario study. For example, (i) the daily water intake and dermal contact of target population groups are not measured during the groundwater sampling, (ii) body weights of the local population are not evaluated (instead, the representative data of the Indian Council of Medical Research (ICMR) and USEPA are used), (iii) average time, dermal permeability and conversion factor are considered as the same, fixed or similar values for deterministic and probabilistic approaches for different age groups, (iv) the variables data to generate the probability distribution functions (PDFs) using MCS are acquired from the USEPA and other relevant published literatures, (v) assumption that the concentrations of specific chemical parameters in groundwater are fully bio-absorbed in the human body may lead to ambiguity in risk analysis, and finally (vi) the reference doses (*RfD*) for ingestion and dermal exposures are obtained from USEPA.

### Hydrogeochemical processes

**Figure 4:**
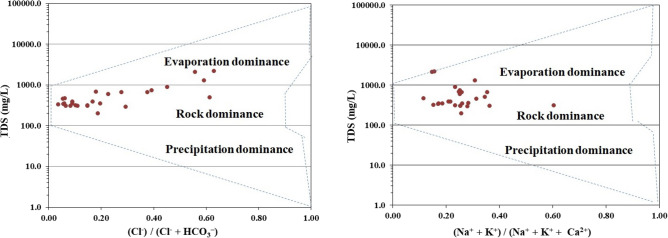
Gibbs diagram representing the factors controlling groundwater chemistry: This diagram enables to understand the relationship between cation ratio [Na^+^/(Na^+^ + Ca_2_^+^)] or anion ratio [Cl^−^/(Cl^−^ + HCO_3_^−^)] versus TDS defining three distinct areas, namely evaporation, rock-water and precipitation zones to elucidate the dominant mechanism influencing the groundwater chemistry of the study area.

Gibbs diagram is applied to elucidate the mechanism controlling groundwater chemistry in the study area^98^. This diagram enables understanding of the relationship between cation ratio [Na^+^/(Na^+^ + Ca^2+^)] or anion ratio [Cl^−^/(Cl^−^ + HCO_3_^−^)] versus TDS, thereby defining three distinct areas, namely evaporation, rock-water and precipitation zones (Fig. 4) that depicts that majority of the groundwater samples (88.89%) are clustered in the rock dominance zone and the remaining samples (11.11%) fall in the evaporation zone.

Groundwater chemistry is primarily influenced by various geochemical processes, especially the interaction of percolating water with subsurface rocks and the chemical solute exchange processes of aquifer minerals in the study area. Many researchers conclude that the elevated concentrations of F^−^ in groundwater are proportionately related to rock-water interaction^99–102^. Besides the rock weathering processes, climatic factors too play a critical role in regulating the evaporation in the semiarid region^26^. The scattering of samples in the Gibbs diagram signifies the impact of anthropogenic inputs in the aquifer systems. The role of the evaporation factor enhances the groundwater salinity by elevating the Na^+^ and Cl^−^ ions, resulting in the higher TDS concentrations, which are further abetted by anthropogenic activities^103^.

**Figure 5:**
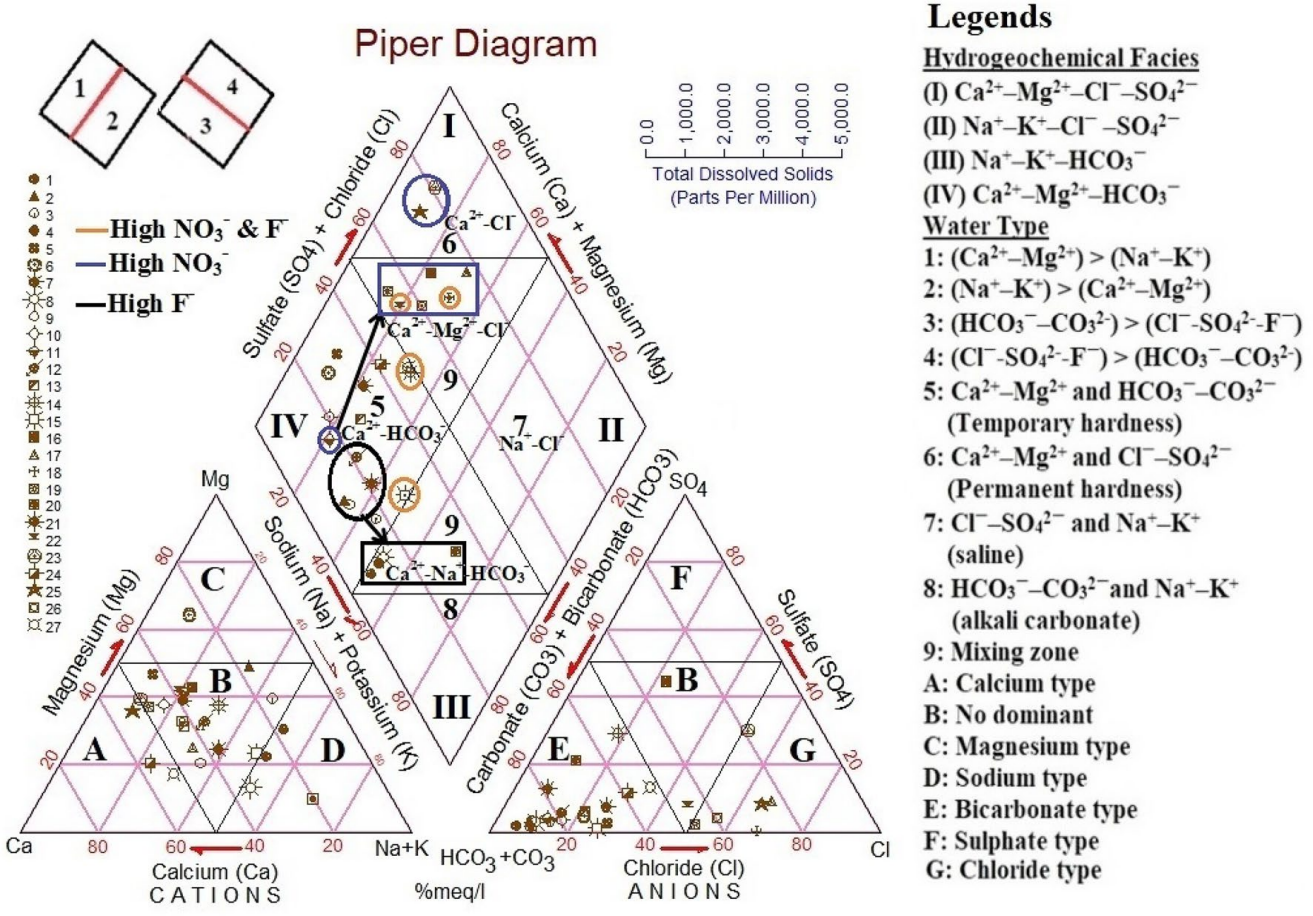
Piper diagram illustrating hydrochemical facies and water types: This graphical interpolation enables characterization of the hydrochemical interaction, genesis of water and groundwater contamination sources. Black arrows signify the conversion of water types due to anthropogenic and geogenic factors.

Piper diagram is a widely used graphical interpolation to characterize the hydrochemical interaction, water genesis and groundwater contamination sources^29,104,105^. Figure 5 depicts that the groundwater is predominantly dominated by alkaline earths over the alkalies and weak acids over the strong acids. This is represented by three hydrochemical facies, namely Ca^2+^–Mg^2+^–HCO_3_^−^ (55.56%), Ca^2+^–Mg^2+^–Cl^−^–SO_4_^2−^ (29.63%) and Na^+^–K^+^–HCO_3_^−^ (14.81%). Also, the groundwater samples are further classified into four water types, i.e., Ca^2+^–HCO_3_^−^ (55.56%), Ca^2+^–Cl^−^ (7.40%), Ca^2+^–Mg^2+^–Cl^−^ (22.22%) and Ca^2+^–Na^+^–HCO_3_^−^ (14.81%). The highest percentage of Ca^2+^–HCO_3_^−^ water type indicates dissolution of carbonate minerals with percolating water from irrigation runoff and precipitation in the subsurface aquifers^11,76^. The cations triangle shows that majority of the samples (70.37%) belong to no-dominant zone, and the remaining samples of 11.11%, 14.82% and 3.70% represent water types in Ca^2+^, Na^+^ and Mg^2+^ dominated zones, respectively. In the anions triangle, around 70.37% samples fall in HCO_3_^−^ water type, which indicates weathering of carbonates and silicates minerals and ion exchange processes in the groundwater^106^. Approximately 22.22% of the samples belonging to Cl^−^ water type depict the role of anthropogenic factors and dissolution of evaporities in the groundwater^26^. The transformation of water types from Ca^2+^–HCO_3_^−^ to Ca^2+^–Cl^−^ and Ca^2+^–Mg^2+^–Cl^−^ types divulges the adverse impacts of human activities and applications of N-chemicals on cultivated lands, thereby elevating the NO_3_^−^ concentrations in groundwater^107,108^. Further, the conversion of water from Ca^2+^–HCO_3_^−^ to Ca^2+^Mg^2+^–Cl^−^ and Ca^2+^–Na^+^–HCO_3_^−^ types is due to the dissolution of fluorite minerals (CaF_2_) and cation exchange between Ca^2+^ and Na^+^^109,110^. Subba Rao suggests that the weathering of rocks, higher Na^+^ and HCO_3_^−^ (or NaHCO_3_) and alkaline nature of water favour the gradual increase of F^−^ concentrations in groundwater^100,111^.

### Source apportionment and geochemical relationships of NO_3_^−^ and F^−^ with other parameters

Many workers have studied the relationship of nitrate and fluoride with specific parameters through scatter plots. For example, for nitrate: NO_3_^−^ versus pH^76^, NO_3_^−^ versus Cl^−17^, NO_3_^−^ versus K^+^, NO_3_^−^ versus Ca^2+^, NO_3_^−^ versus SO_4_^2−^, NO_3_^−^ versus Cl^−^^112^, NO_3_^−^ versus EC, NO_3_^−^ versus Cl^−^, NO_3_^−^ versus K^+^, NO_3_^−^ versus SO_4_^2−^, NO_3_^−^ versus Na^+^, NO_3_^−^ versus Ca^2+^, NO_3_^−^ versus Mg^2+^, NO_3_^−^ versus HCO_3_^−^^27^, and for fluoride: F versus pH, F^−^ versus HCO_3_^−^^76^, F^−^ versus HCO_3_^−^, F^−^ versus Na^+^, F^−^ versus NO_3_^−^^102^, F^−^ versus pH, F^−^ versus Ca^2+^^113^, F^−^ versus pH, F^−^ versus Na^+^, F^−^ versus K^+^, F^−^ versus HCO_3_^−^, F^−^ versus Ca^2+^^114^. However, these studies have not evaluated NO_3_^−^ and F^−^ holistically for their geochemical relationships with physical parameters and major cations and anions and also their source apportionment with site-specific datasets available. The present study is unique in the sense that it uses scatter plots to correlate NO_3_^−^ and F^−^ with other physicochemical parameters independently (pH, EC, TH, Ca^2+^, Mg^2+^, Na^+^, K^+^, Cl^−^, HCO_3_^−^, SO_4_^2−^, and F^−^ versus NO_3_) to achieve these objectives.

### Source apportionment and geochemical relationship of NO_3_^−^ with other parameters

**Figure 6:**
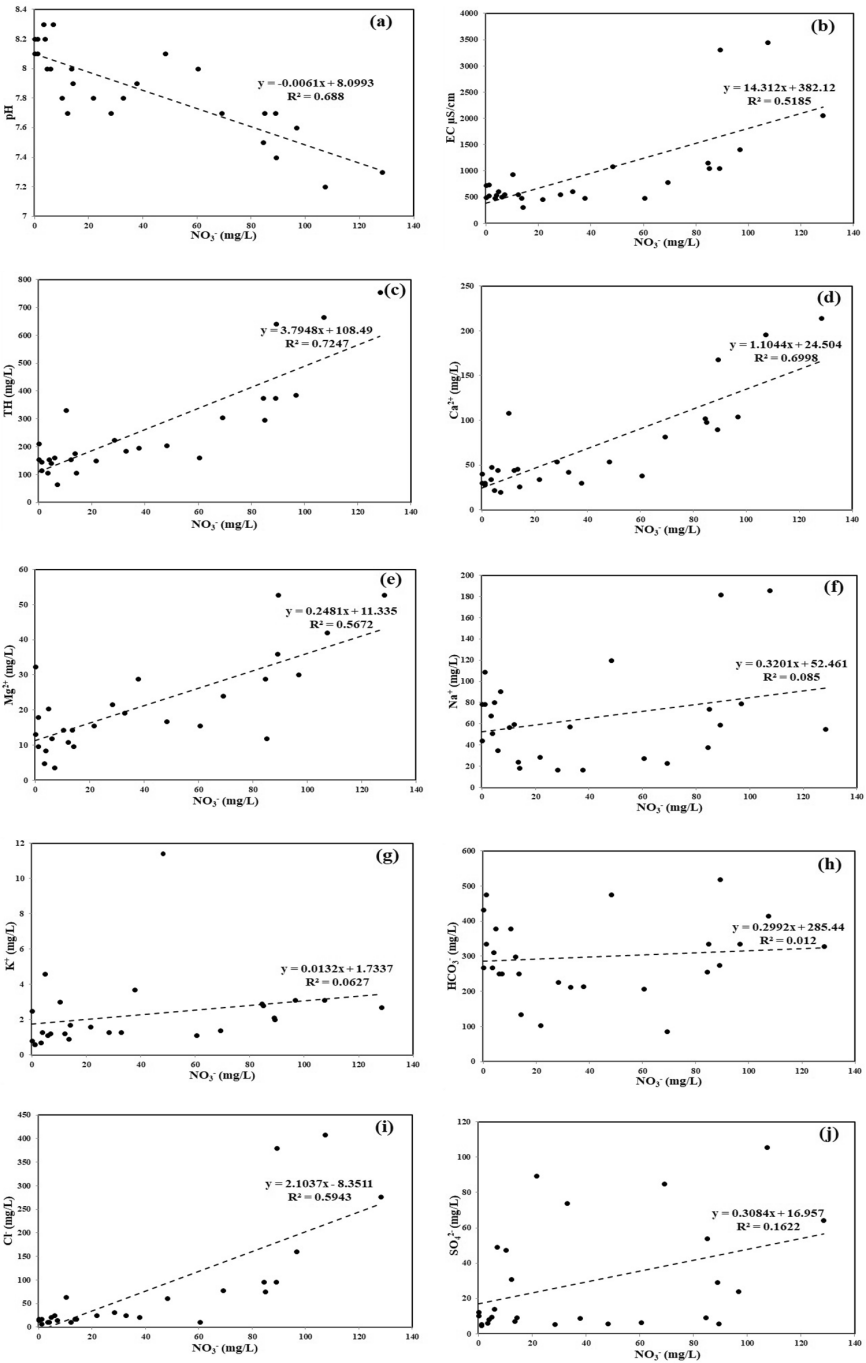
Scatter plot correlations between NO_3_^−^ and (**a**) pH, (**b**) EC, (**c**) TH, (**d**) Ca^2+^, (**e**) Mg^2+^, (**f**) Na^+^, (**g**) K^+^, (**h**) HCO_3_^−^, (**i**) Cl^−^, (**j**) SO_4_^2−^ in groundwater samples: Each plot signifies the relationship of NO_3_^−^ with a particular physicochemical parameter to understand their geochemical interaction. Source apportionment of NO_3_^−^ is carried out with the help of such interactions.

A strong inverse correlation between NO_3_^−^ and pH (r^2^ = 0.688 and y =  − 0.0061x + 8.0993) indicates decreasing pH values with increasing NO_3_^−^ concentrations (Fig. 6a). Dadgar and Payandeh^115^ too report this relationship in Tabriz province, Iran. The oxidation of dissolved CO_2_ in groundwater forms carbonic acid and readily dissociates into H^+^ and HCO_3_^−^ ions is an intensive process^24^. Further, NO_3_^−^ ions rapidly react with free H^+^ ions to form HNO_3_ resulting in acidic conditions at higher NO_3_^−^ concentrations (Eq. 7).

7 $$\left. \begin{gathered} {\text{CO}}_{2} + {\text{H}}_{2} {\text{O}} \to {\text{H}}_{2} {\text{CO}}_{3} \left( {{\text{Carbonic}}\;{\text{acid}}} \right) \hfill \\ {\text{H}}_{2} {\text{CO}}_{3} \to {\text{H}}^{ + } + {\text{HCO}}_{3}^{ - } \hfill \\ {\text{NO}}_{3}^{ - } + {\text{H}}^{ + } \leftrightarrow {\text{HNO}}_{3} \left( {{\text{Nitric}}\;{\text{acid}}} \right) \hfill \\ \end{gathered} \right\}$$

The scatter plot of NO_3_^−^ versus EC shows a positive correlation (r^2^ = 0.5185), divulging higher mineralization of dissolved substances, including excess NO_3_^−^ concentrations in groundwater (Fig. 6b). The samples with NO_3_^−^ contents above the guideline value of 45 mg/L^46^ have higher EC in groundwater. Such a relationship is often associated with anthropogenic inputs, such as agricultural runoff, domestic sewage, poultry farming and unplanned urbanization, which release an enormous quantity of organic nitrogen and ammonia^76,116–118^. Ammonia is affectively absorbed in the soil particles that restrict its movement. During the limited aerobic condition in the soil, the nitrification process converts the immobilized ammonia into nitrate by bacterial activities, as shown in Eq. (8). Anthropogenic inputs accelerate the nitrification process that enhances easy leaching of NO_3_^−^ from the soil in the percolating water recharging the aquifers.

8 $$\left. \begin{gathered} \mathop {2{\text{NH}}_{3} + 3{\text{O}}_{2} }\limits_{{({\text{Ammonia}})}} \to \mathop {2{\text{NO}}_{2}^{ - } }\limits_{{({\text{Nitrite}})}} + 2{\text{H}}^{ + } + 2{\text{H}}_{2} {\text{O}} \hfill \\ 2{\text{NO}}_{2} + {\text{O}}_{2} \to 2{\text{NO}}_{3}^{ - } \left( {{\text{Nitrate}}} \right) \hfill \\ \end{gathered} \right\}$$

The possible mineral source contributing Ca^2+^ and Mg^2+^ in the groundwater is determined by Ca^2+^/Mg^2+^ ratio^119^. Figure 6d,e depicts the positive relationship of NO_3_^−^ with Ca^2+^ (r^2^ = 0.6998) and Mg^2+^ (r^2^ = 0.5672), which indicates cation exchange processes in the groundwater due to prolonged application of N-fertilizers for crop production^120^. This cation exchange process significantly enhances the mineralization of Ca^2+^ and Mg^2+^ and elevates NO_3_^−^ concentrations. Also, the nitrification process increases the NO_3_^−^ level and acidity in groundwater resulting in Ca^2+^ and Mg^2+^ enrichment by the dissolution of carbonate minerals^76,121^, as illustrated in Fig. S3a, i.e., 14.8% samples by dolomite and 48.2% by calcite in our study area. The remaining 37% samples have Ca^2+^/Mg^2+^ ratio values > 2 depicting the influence of silicate weathering in groundwater^122^. The study area is a metamorphic terrain with a rich deposition of calcsilicate, hornblende, quartz and biotite^57^. Therefore, Ca^2+^ and Mg^2+^ concentrations in groundwater are influenced by carbonate and silicate rock-water interaction as expressed in Eqs. (9)–(12)^24,123–125^.

9 $${\text{CaCO}}_{3} + {\text{H}}_{2} {\text{CO}}_{3} \leftrightarrow {\text{Ca}}^{2 + } + 2{\text{HCO}}_{3}^{ - } \left( {{\text{calcite}}\;{\text{dissolution}}} \right)$$

10 $${\text{CaMg}}({\text{CO}}_{3} )_{2} + 2{\text{H}}^{ + } \leftrightarrow {\text{CaCO}}_{3} + {\text{Mg}}^{2 + } + {\text{H}}_{2} {\text{CO}}_{3} \left( {{\text{dolomite}}\;{\text{dissolution}}} \right)$$

11 $${\text{CaSO}}_{4} + {\text{CaMgCO}}_{3} + 6{\text{H}}^{ + } \leftrightarrow {\text{CaCO}}_{3} + {\text{Ca}}^{2 + } + {\text{Mg}}^{2 + } + {\text{SO}}_{4}^{2 - } + {\text{H}}_{2} {\text{CO}}_{3} \left( {{\text{Anhydrite}}\;{\text{and}}\;{\text{dolomite}}\;{\text{dissolution}}} \right)$$

12 $$\left( {{\text{Na}},{\text{K}},{\text{Ca}},{\text{Mg}}} \right)\;{\text{silicate}} + {\text{H}}_{2} {\text{CO}}_{3} \to {\text{Na}}^{ + } + {\text{K}}^{ + } + {\text{Ca}}^{2 + } + {\text{Mg}}^{2 + } + {\text{H}}_{4} {\text{SiO}}_{4} + {\text{HCO}}_{3}^{ - } + {\text{Clay}}$$

Since NO_3_^−^ has a strong positive loading with Ca^2+^ and Mg^2+^, it exhibits a significant positive correlation with TH (r^2^ = 0.7247) (Fig. 6c). Water hardness is attributed to the elevated concentrations of dissolved alkaline earth elements (Ca^2+^ and Mg^2+^) in the aquifer system^79^. The scatter plots of NO_3_^−^ with Na^+^ (r^2^ = 0.085) (Fig. 6f) and K^+^ (r^2^ = 0.0627) (Fig. 6g) signify a very weak positive relationship and suggest that the anthropogenic inputs are not the only primary source of alkali ions contents in groundwater. The bivariate plot of Na^+^ + K^+^ versus TZ^+^ (Fig. S3b) depicts that the entire groundwater samples fall below the 1:1 aquiline. This indicates the weathering effect of silicate minerals besides the anthropogenic impacts, such as the application of NPK fertilizers and discharge of untreated sewerage water on the open ground, which elevate the Na^+^ and K^+^ concentrations in groundwater^11,27,126,127^. In the study area, albite, microcline and alunite dissolution are the key sources of Na^+^ and K^+^ ions through rock-water interactions, as shown in Eqs. (13)–(15).

13 $$\mathop {2{\text{NaAlSi}}_{3} {\text{O}}_{8} }\limits_{{({\text{Albite}})}} + 2{\text{CO}}_{2} + 11{\text{H}}_{2} {\text{O}} \to \mathop {{\text{Al}}_{2} {\text{Si}}_{2} {\text{O}}_{5} \left( {{\text{OH}}} \right)_{4} }\limits_{{({\text{Kaolinite}})}} + 4{\text{H}}_{4} {\text{SiO}}_{4} + 2{\text{Na}}^{ + } + 2{\text{HCO}}_{3}^{ - }$$

14 $$\mathop {2{\text{KAlSi}}_{3} {\text{O}}_{8} }\limits_{{({\text{Microcline}})}} + 2{\text{CO}}_{2} + 11{\text{H}}_{2} {\text{O}} \to \mathop {{\text{Al}}_{2} {\text{Si}}_{2} {\text{O}}_{5} \left( {{\text{OH}}} \right)_{4} }\limits_{{({\text{Kaolinite}})}} + 4{\text{H}}_{4} {\text{SiO}}_{4} + 2{\text{K}}^{ + } + 2{\text{HCO}}_{3}^{ - }$$

15 $$\mathop {{\text{KAl}}_{3} ({\text{SO}}_{4} )_{2} \left( {{\text{OH}}} \right)_{6} }\limits_{{({\text{Alunite}})}} + 3{\text{CO}}_{2} + {\text{H}}_{2} {\text{O}} \to \mathop {3{\text{Al}}\left( {{\text{OH}}} \right)_{3} }\limits_{{({\text{Gibbsite}})}} + 3{\text{HCO}}_{3}^{ - } + {\text{K}}^{ + } + 2{\text{SO}}_{4}^{2 - }$$

The scatter plot of NO_3_^−^ versus HCO_3_^−^ shows the least positive loading (r^2^ = 0.012) among the anions (Fig. 6h). This relationship suggests that the HCO_3_^−^ does not exhibit much variation with increasing or decreasing NO_3_^−^ concentrations. The fact that HCO_3_^−^ ions are the dominant anions in the groundwater samples confirms that its primary source is possibly carbonate and silicate weathering^26,76,122,128^, as shown in Eqs. (9)–(12).

In NO_3_^−^ versus Cl^−^ plot (Fig. 6i), their positive correlation (r^2^ = 0.5943) implies a common source, such as a combination of oxidation of animal and human waste^44^, application of manure and nitrogenous fertilizers^129^, septic tank seepages^130^, agricultural runoff^131^, etc. Similar findings are reported in the semiarid regions of many Indian States, such as Punjab^24^, Rajasthan^132^, Andhra Pradesh^133^, and Telangana^134^.

Figure 6j depicts the weak positive loading between NO_3_^−^ and SO_4_^2−^ (r^2^ = 0.1622) due to two separate sets of NO_3_^−^ and SO_4_^2−^ concentrations in the groundwater samples. The samples having low or high NO_3_^−^ levels have both low and high SO_4_^−^ concentrations, thus neglecting the influence of the anthropogenic activities on SO_4_^2−^.

The plot of Ca^2+^ versus SO_4_^2−^ (Fig. S3c) is meant to identify the minerals that contribute to higher amount of Ca^2+^ and SO_4_^2−^ ions in groundwater^76^. Majority of the samples (92.6%) are below the equiline (1:1), indicating that the role of gypsum (CaSO_4_·2H_2_O) dissolution is insignificant. The remaining samples (7.4%) falling along the equiline depict the dissolution of anhydrite (CaSO_4_) mineral in the groundwater^135,136^. The gypsum precipitation in the groundwater occurs through direct hydration of anhydrite and dissolution of calcium-bearing minerals oxidized with sulphate and hydronium ions^137^, as expressed in Eqs. (16) and (17). Hence, the weak positive correlation between Ca^2+^ and SO_4_^2−^ (r^2^ = 0.197) (Fig. S3c) suggests that the limited concentrations of Ca^2+^ ions in the groundwater may be due to the precipitation of gypsum^138^. If the study area lacks gypsum mineral, then the biologically oxidized sulphur containing compounds deposited by the rainwater and nitrogen compounds in the soil leach down to groundwater as SO_4_^2−^ and NO_3_^−^ ions^139^. Thus, the positive regression line between NO_3_^−^ and SO_4_^2−^ (y = 0.3084x + 16.957) (Fig. 6j) is found in the groundwater samples of the study area. Karunanidhi^27^ report similar findings on the positive relationship between NO_3_^−^ and SO_4_^2−^ in the groundwater samples of Tiruppur region, India. Moreover, the dissolution of alunite [KAl_3_(SO_4_)_2_(OH)_6_], as expressed in Eq. (15), will also contribute to the SO_4_^2−^ ions in groundwater.

16 $${\text{CaSO}}_{4} + 2{\text{H}}_{2} {\text{O}} \to {\text{CaSO}}_{4} \cdot 2{\text{H}}_{2} {\text{O}}$$

17 $${\text{CaCO}}_{3} + 2{\text{H}}^{ + } + {\text{SO}}_{4}^{2 - } + {\text{H}}_{2} {\text{O}} \to {\text{CaSO}}_{4} \cdot 2{\text{H}}_{2} {\text{O}} + {\text{CO}}_{2}$$

### Source apportionment and geochemical relationship of F^−^ with other parameters

**Figure 7:**
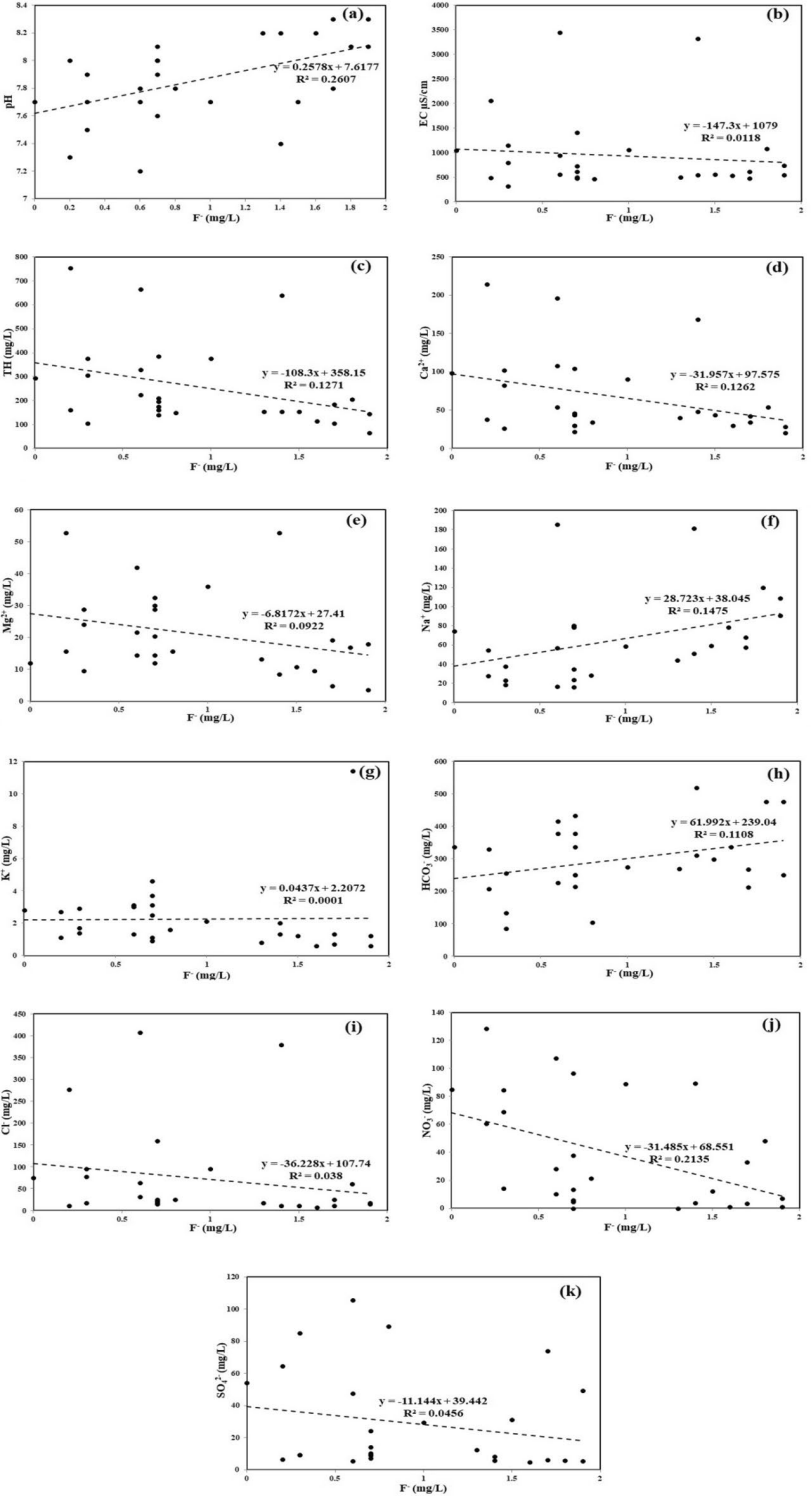
Scatter plot correlations between F^−^ and (**a**) pH, (**b**) EC, (**c**) TH, (**d**) Ca^2+^, (**e**) Mg^2+^, (**f**) Na^+^, (**g**) K^+^, (**h**) HCO_3_^−^, (**i**) Cl^−^, (**j**) NO_3_^−^, (**k**) SO_4_^2−^ in groundwater samples: Each plot signifies the relationship of F^−^ with a particular physicochemical parameter to understand their geochemical interaction. Source apportionment of F^−^ is carried out with the help of such interactions.

Normally, high pH in groundwater depicts its alkaline nature, resulting in elevated concentrations of HCO_3_^−^ and high hydroxyl (OH^−^) ions (Eq. 18, Tables 2 and S2, Fig. S2). A fairly positive relationship between pH and F^−^ (r^2^ = 0.2607; Fig. 7a) indicates that the alkaline water favours dissolution and mobilization of F^−^ bearing minerals in groundwater^140^. The weathering processes of fluoride-bearing rocks to replace F^−^ ions with OH^−^ ions in the lattices of different minerals, namely muscovite, biotite, amphibole, and hornblende, has enriched the F^−^ concentrations in groundwater. Xiao^141^ and Karunanidhi^142^ express the displacement mechanism of F^−^ ions by OH^−^ ions in the muscovite, biotite, and hornblende minerals as follows (Eqs. 19–21).

18 $${\text{HCO}}_{3}^{ - } + {\text{H}}_{2} {\text{O}} = {\text{H}}_{2} {\text{CO}}_{3} + {\text{OH}}^{ - }$$

19 $${\text{KAl}}_{2} \left[ {{\text{AlSi}}_{3} {\text{O}}_{10} } \right]{\text{F}}_{2} + 2{\text{OH}}^{ - } = {\text{KAl}}_{2} \left[ {{\text{AlSi}}_{3} {\text{O}}_{10} } \right]\left( {{\text{OH}}} \right)_{2} + 2{\text{F}}^{ - }$$

20 $${\text{KMg}}_{3} \left[ {{\text{AlSi}}_{3} {\text{O}}_{10} } \right]{\text{F}}_{2} + 2{\text{OH}}^{ - } = {\text{KMg}}_{3} \left[ {{\text{AlSi}}_{3} {\text{O}}_{10} } \right]\left( {{\text{OH}}} \right)_{2} + 2{\text{F}}^{ - }$$

21 $${\text{NaCa}}_{2} ({\text{Mg}},{\text{Fe}},{\text{Al}})_{5} ({\text{Al}},{\text{Si}})_{8} {\text{O}}_{22} {\text{F}}_{2} + 2{\text{OH}}^{ - } \to {\text{NaCa}}_{2} ({\text{Mg}},{\text{Fe}},{\text{Al}})_{5} ({\text{Al}},{\text{Si}})_{8} {\text{O}}_{22} \left( {{\text{OH}}} \right)_{2} + 2{\text{F}}^{ - }$$

Figure 7b shows a negative trend between the EC and F^−^ (r^2^ = 0.0118; y = -147.3x + 1079), indicating no major influence of EC on F^−^ ion concentrations. A rather weak, but negative relationship of F^−^ with Ca^2+^ (r^2^ = 0.1262, y = -31.957x + 97.575) and Mg^2+^ (r^2^ = 0.0922, y = -6.8172x + 27.41) indicates decreasing concentrations of Ca^2+^ and Mg^2+^ ions in groundwater with increasing F^−^ content (Fig. 7d,e). Various workers report similar findings between F^−^ versus Ca^2+^ elsewhere^53,110^. The excess concentrations of HCO_3_^−^ with high pH contribute to the alkaline water, thus favouring the dissolution of fluorite (CaF_2_) in groundwater due to precipitation of CaCO_3_^143^, as shown in Eq. (22).

22 $${\text{CaF}}_{2} + 2{\text{HCO}}_{3}^{ - } = {\text{CaCO}}_{3} + 2{\text{F}}^{ - } + {\text{H}}_{2} {\text{O}} + {\text{CO}}_{2}$$

Jack suggests that the rock-water interaction of fluoride-bearing minerals from a recharge area through the facture zone would precipitate the Ca^2+^ and Mg^2+^ ions as calcite, Mg-Calcite dolomite, and dolomite fluorite, respectively, along the groundwater flow path to a discharge area^144^. Hem states that because Ca^2+^ and Mg^2+^ ions are divalent cations with similar properties, they possess the same stability with other ion pairs (SO_4_^2−^, CO_3_^2−^ and HCO_3_^−^) and contribute similarly to water hardness^145^. Thus, the inverse relationship between TH and F^−^ (Fig. 7c) is due to decreased Ca^2+^ and Mg^2+^ ion concentrations or precipitation of calcium carbonate and Mg-calcite dolomite causing enhanced solubility of fluoride-bearing minerals in the study area^139,146^.

While examining the role of Na^+^, it is found that the Na^+^/Ca^2+^ ratio helps in understanding the probable reason for lowering of Ca^2+^ activity in groundwater^144^. Around 37% of water samples have Na^+^/Ca^2+^ ratio > 1, indicating that evapotranspiration is possibly affecting the Ca^2+^ activity by precipitating it and increasing the Na^+^ concentrations, thus favouring the enrichment of F^−^ content in groundwater (Fig. S3d). The study area is a semiarid region characterized by drier climatic conditions where the dissolved constituents are readily concentrated and precipitated by evaporation, thereby leading to groundwater salinity^147,148^. The remaining 63% groundwater samples show Na^+^/Ca^2+^ ratio < 1, which depicts that rock-water interaction is another key contributing factor of generation of Ca^2+^ and F^−^ ions due to the dissolution of fluorite minerals in the groundwater. However, Ca^2+^ ions subsequently react with NaHCO_3_ to form CaCO_3_ precipitation (Eq. 23). In a similar line, Arveti^99^ report that high F^−^ content in groundwater is directly related to the dissolution of fluoride enriched minerals due to prolonged residence time of water due to physiographic conditions or low hydraulic conductivity in aquifers providing a longer contact period. The plot Na^+^ versus F^−^ (r^2^ = 0.1475) with a positive slope (y = 28.723x + 38.045) indicates gradual increase of F^−^ concentrations with elevated Na^+^ content in groundwater (Fig. 7f). The higher concentrations of NaHCO_3_ or Na^+^ ions with alkaline pH in groundwater allows dissolution of F^−^ ions from fluorite (CaF_2_) through rock–water interaction^143,149^ (Eq. 23).

23 $${\text{CaF}}_{2} + 2{\text{NaHCO}}_{3} = {\text{CaCO}}_{3} + 2{\text{Na}}^{ + } + 2{\text{F}}^{ - } + {\text{H}}_{2} {\text{O}} + {\text{CO}}_{2}$$

In Fig. 7g, the plot K^+^ versus F^−^ divulges that there is no significant positive or negative relationship between them. The flat linear regression (r^2^ = 0.0001) indicates that the K^+^ does not have much influence on the fluoride mineralization in groundwater. The orthoclase feldspar (KAlSiO_3_O_8_) is generally resistant to attack by water, but apparently gets altered to silica, clay, and K^+^ ions^145^. In the study area, rapid precipitation of alunite occurs in the aquifers due to the high degree of stability of potassium-bearing alumino-silicate minerals resulting in low content of K^+^ in groundwater.

The positive trend between HCO_3_^−^ and F^−^ (r^2^ = 0.1108; y = 61.992x + 239.04) divulges that the increase in HCO_3_^−^ content supports the dissolution of F^−^ bearing minerals in groundwater (Fig. 7h). However, few samples have low HCO_3_^−^ concentrations with high F^−^ values which indicates that the F^−^ enrichment in groundwater is affected by a combination of processes, such as evapotranspiration and calcite precipitation^150^. The HCO_3_^−^/Ca^2+^ratio predicts the likelihood of F^−^ enrichment in groundwater^140^. About 85% of samples show HCO_3_^−^/Ca^2+^ratio > 1 (Fig. S3e), signifying that groundwater hydrological conditions are still favourable for further enrichment of fluoride minerals in the study area with their saturation index prevailing in the order of -2.66 to -0.68 (undersaturated condition).

The application of phosphatic and chloride containing fertilizers are the main anthropogenic sources of high F^−^, NO_3_^−^ and Cl^−^ contamination in the groundwater^24,150^. Figure 7i,j shows inverse relationship of F^−^ with Cl^−^ (r^2^ = 0.038, y = -36.228x + 107.74) and NO_3_^−^ (r^2^ = 0.2135, y = -31.485x + 68.551), respectively. These plots signify that F^−^ contamination in groundwater is from a different source than that of Cl^−^ and NO_3_; thus, the role of agricultural inputs for F^−^ generation is neglected. In some cases, when the redox potential falls below a certain value in groundwater, the denitrification process of NO_3_^−^ by the nitrate-reducing bacteria, accompanied by increased pH value, enhances the precipitation of Ca^2+^ resulting in the high F^−^ and HCO_3_^−^ concentrations in water (Eq. 24)^139^.

The inverse correlation between SO_4_^2−^ and F^−^ (r^2^ = 0.0456; y =  − 11.144x + 39.442) indicates two different sets of SO_4_^2−^ and F^−^ contents in the groundwater samples (Fig. 7k). The samples having low or high F^−^ levels have both low and high SO_4_^−^ concentrations, thus neglecting the influence of anthropogenic activities. In groundwater, when the redox potential is below a specific value due to high evapotranspiration, sulphate-reducing bacteria initiate desulphurisation process that results in the loss of SO_4_^2−^ ions (Eq. 25). Further, the desulphurisation process raises the pH value, thus favouring the fluorite solubility leading to the high concentrations of F^−^ and HCO_3_^−^ ions and precipitation of Ca^2+^ ions as CaCO_3_ in groundwater^139^. Many researchers have observed similar relationship between SO_4_^2−^ versus F^−^ elsewhere^144,150,151^, because the decrease in solubility of fluorite minerals is affected by the presence of SO_4_^2−^ ions in groundwater.

24 $$2{\text{NO}}_{3} \to \mathop {\mathop {2{\text{HO}}}\limits_{ \downarrow } }\limits_{{{\text{NH}}_{3} }} - {\text{N}} = {\text{O}} \to {\text{HO}} - {\text{N}} = {\text{N}} - \mathop {\mathop {{\text{OH}}}\limits_{ \searrow } \to \mathop {N_{2} }\limits_{ \nearrow } }\limits_{{{\text{N}}_{2} {\text{O}}}}$$

25 $${\text{SO}}_{4}^{2 - } + {\text{CH}}_{4} \to {\text{HS}}^{ - } + {\text{HCO}}_{3}^{ - } + {\text{H}}_{2} {\text{O}}$$

### Chemometric analysis

#### Principal component analysis

Principal component analysis (PCA) is applied on the 13 chemical parameters to extract the significant principal components (PCs) that define the hydrogeochemistry in the study area and help in identifying the probable sources of these parameters in groundwater. A scree plot is generated to determine the eigenvalues of the PCs using the varimax rotation method. Three PCs were considered as significant from the entire extracted PCs whose eigenvalues are greater than 1. The eigenvalue of PC1, PC2 and PC3 are 57.60, 18.60 and 9.90, respectively, and their cumulative variance is 86.10% of all analyzed parameters (Table 5). The significant PCs having parameters loading scores of > 0.75 (strong, marked bold) and between 0.50 and 0.75 (moderate, marked bold with italics) are considered for the PCA interpretation. The first principal component (PC1) that explains 57.60% of the cumulative variance shows strong positive loading on EC, TDS, TH, Ca^2+^, Mg^2+^, Cl^−^ and NO_3_^−^ and a strong inverse relationship with pH (Table 5).

**Table 5:** Rotated varimax component matrix of the analysed groundwater samples around village Supebeda in Chhattisgarh State, India.

Variable	PC1	PC2	PC3	Communality
pH	** − 0.92**	0.17	0.08	0.89
EC	**0.89**	0.42	0.03	0.98
TDS	**0.89**	0.42	0.03	0.98
TH	**0.98**	0.09	0.04	0.96
Ca^2+^	**0.96**	*0.10*	− 0.02	0.93
Mg^2+^	**0.86**	0.06	0.17	0.78
Na^+^	0.45	**0.85**	0.13	0.94
K^+^	0.18	0.08	**0.79**	0.67
HCO_3_^−^	0.26	***0.74***	***0.50***	0.87
Cl^−^	**0.93**	0.31	− 0.02	0.97
SO_4_^2−^	0.49	− 0.09	*** − 0.59***	0.59
NO_3_^−^	**0.90**	− 0.17	0.09	0.85
F^−^	− 0.45	**0.77**	− 0.07	0.81
Eigen values	7.48	2.41	1.29	11.19
% of variance	57.60	18.60	9.90	
Cumulative % of variance	57.60	76.20	86.10	
Probable identified sources	Mixed factors (lithogenic and anthropogenic inputs)	Fluoride dissolution through rock-water interaction	Weathering of bedrocks, evapotranspiration and groundwater extraction	

Bold indicates strong loading between parameters. Bold-Italics indicates moderate loading between parameters.

*PC* Principal component.

The loading TH (0.98) is directly related to Ca^2+^ (0.96) and Mg^2+^ (0.86) scores that indicate that water hardness is influenced by the alkaline earths concentrations in aquifers^77,104^. The weathering and dissolution of carbonate (calcite and dolomite) and silicate minerals through rock-water interaction are the probable sources of Ca^2+^ and Mg^2+^ in groundwater, which is also supported by Ca^2+^/Mg^2+^ ratio^24^. The weak loading of alkalis (Na^+^: 0.45 and K^+^: 0.18) with respect to alkaline earths (Ca^2+^ and Mg^2+^) supports the cation ion exchange process in groundwater^79^.

Both Cl^−^ (0.93) and high loading of NO_3_^−^ (0.90) indicate the effect of agrochemicals and domestic sewage in groundwater^29,152^. The application of chemical fertilizers, namely anhydrous ammonium chloride, ammonium nitrate and urea containing inorganic chlorine and nitrogen, is a matter of concern^11^. The inverse loading of pH (-0.92) is due to the oxidation of dissolved CO_2_ and organic matter forming carbonic acids, thereby releasing free H^+^ ions^153^. The inorganic chlorine and nitrogen react with H^+^ ions rapidly to form HCl and HNO_3_, which decrease pH in groundwater. The high scores of EC (0.89) and TDS (0.89) are due to the elevated concentrations of Ca^2+^, Mg^2+^, Cl^−^ and NO_3_^−^ ions, which enhance the mineralization of groundwater in the study area. Therefore, PC1 is controlled by lithogenic (Ca^2+^ and Mg^2+^) and anthropogenic (Cl^−^ and NO_3_^−^) factors.

The second principal component (PC2) explains 18.60% of the total variance. It is positively weighed on Na^+^ (0.85) and F^−^ (0.77), moderately weighed on HCO_3_^−^ (0.74) and has insignificant loading on Ca^2+^ (0.10) indicating lithogenic sources of these elements (Table 5). PC2 indicates that the dissolution of fluoride-bearing minerals is influenced by the elevated concentrations of Na^+^ and HCO_3_^−^ or NaHCO_3_^−^ in the aquifer system. On the other hand, the weak correlation of Ca^2+^ with F^−^ (Fig. 7d) suggests that high Ca^2+^ content in groundwater inhibits fluoride mineralization at alkaline pH^25,154,155^). Therefore, PC2 deals with fluoride dissolution through rock-water interaction.

Lastly, in the principal component 3 (PC3), a variance of 9.90% depicts positive correlation with K^+^ (high: 0.79) and HCO_3_^−^ (moderate: 0.50), and negative loading on SO_4_^2−^ (moderate: − 0.59) (Table 5). The main sources of K^+^ and HCO_3_^−^ are the weathering of silicate, muscovite, biotite, and microcline minerals found in the study area. The negative score of SO_4_^2−^ is due to the leaching of inorganic sulphides present in the sediments through percolating water, weathering of pyrite-sulphides bearing minerals, namely pyroxene, amphiboles, magnetite and olivine^156^ and biological oxidation of sulphur containing compounds in soil^139^. The oxidation of these minerals present in the soil profile or subsurface layers is operated through oxygen transport, viz., convection process and direct exposure of air, because of lowering of groundwater levels through evapotranspiration and groundwater extraction^157^. Further, the inverse correlations of SO_4_^2−^ with K^+^ and HCO_3_^−^ reflect the different minerals sources contributing to these ions in the aquifer system. The concentrations of K^+^, HCO_3_^−^ and SO_4_^2−^ are well within the acceptable limits or guideline values of BIS^46^ and WHO^45^, thus indicating geogenic inputs.

### Cluster analysis

**Figure 8:**
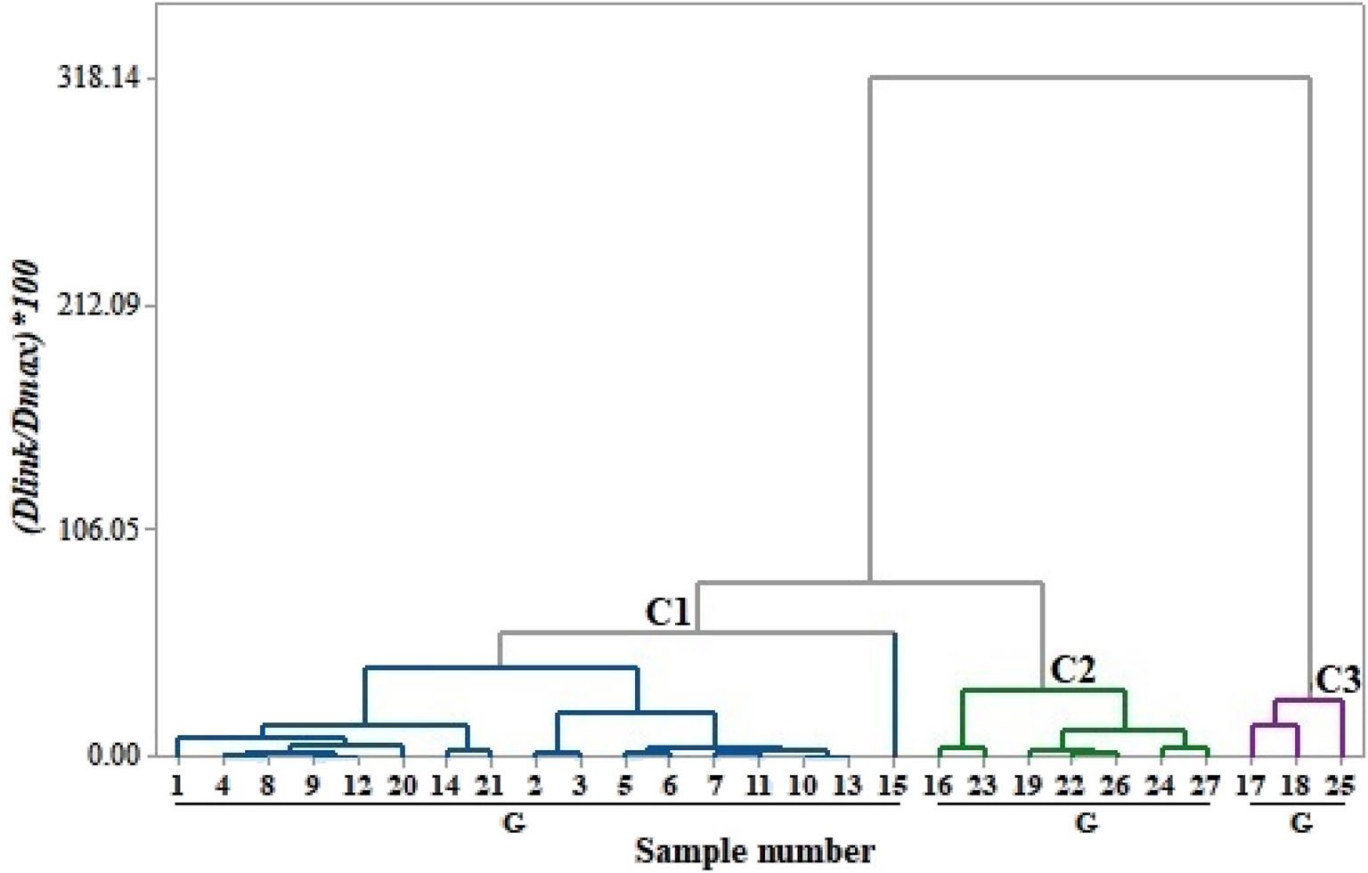
Dendrogram of groundwater sampling locations around village Supebeda in Chhattisgarh State, India: Three different clusters (C1, C2, and C3) are identified by Ward's method and the Euclidean distance to determine the similarity/dissimilarity. The relatively homogenous samples are grouped in each cluster and marked by certain specific parameters controlling them. In the y-axis, (*D*_*limk*_/*D*_*max*_) * 100 represents the quotient between the linkage distances for a particular case divided by the maximal linkage distance. The quotient is then multiplied by 100 to standardize the linkage distance represented by the y-axis.

Cluster analysis (CA) is employed on the 27 groundwater samples to create different clusters by grouping similar samples in the form of a dendrogram. The samples grouped in each cluster are marked by certain specific parameters controlling them. Therefore, the variation in the clusters can be identified by computing the average value of each parameter of the sample(s) within a cluster to assess the specific tracers for each cluster^82,86^. Figure 8 depicts three significant clusters [(*D*_*limk*_/*D*_*max*_) * 100 < 105] from the dendrogram. Table 6 provides the average values of the groundwater parameters for each cluster. Cluster 1 (C1) is formed by the largest number of samples (G1, G4, G8, G9, G12, G20, G14, G21, G2, G3, G5, G6, G7, G10, G11, G13, G15) with highest values of pH and F^−^, higher values of Na^+^ and HCO_3_^−^ and lowest value of Ca^2+^ that indicate fluoride enrichment. The average values of the parameters belonging to C1 are below their respective standard limits of BIS^46^ and WHO^45^, except for F^−^ (1.14) (Table 6). Thus, the groundwater quality of C1 is influenced by the dissolution of fluoride-bearing minerals and fits well with PC2.

**Table 6:** Average values of the physicochemical parameters for each cluster.

Parameters	C1	C2	C3
pH	**8.03**	7.69	*7.30*
EC	572.00	980.43	**2939.67**
TDS	366.08	***627.47***	**1881.39**
TH	156.18	***316.43***	**686.67**
Ca^2+^	*37.06*	***88.29***	**192.67**
Mg^2+^	15.25	22.97	**49.20**
Na^+^	***57.29***	51.17	**140.57**
K^+^	2.12	***2.41***	**2.60**
HCO_3_^−^	***293.49***	252.86	**421.00**
Cl^−^	19.66	84.73	**355.03**
SO_4_^2−^	15.82	***48.33***	**58.57**
NO_3_^−^	16.10	***65.11***	**108.27**
F^−^	**1.14**	*0.53*	0.73

Bold indicates the highest average value of a parameter among the three clusters. Bold-Italics indicates the second highest average value of a parameter to identify the special tracer. Italics indicates the lowest average value of a parameter among the three clusters.

Cluster 2 (C2) denotes the higher values of TDS, TH, Ca^2+^, K^+^, SO_4_^2−^and NO_3_^−^ and the lowest value of F^−^. The average values of TDS (627.47), TH (316.43), Ca^2+^ (88.29) and NO_3_^−^ (65.11) are above the acceptable limits of BIS^46^ due to their excess concentrations in samples G19, G22, G23, G24, G26, and G27 (Tables S2 and Table 6). The lowest value of F^−^ (0.53) in C2 among the three significant clusters are due to only one sample (G22) that just touches the BIS^46^ acceptable limit of F^−^ (Table S2). The groundwater samples (G16, G23, G19, G22, G26, G24, G27) that represent the C2 have K^+^ and SO_4_^2−^ concentrations below their respective guideline values of WHO^45^ and BIS^46^. Therefore, C2 is influenced by both geogenic and anthropogenic factors.

Finally, C3 is the smallest cluster (G17,G18,G25) and is marked by the highest values of EC, TDS, TH, Ca^2+^, Mg^2+^, Na^+^, K^+^, HCO_3_^−^, Cl^−^, SO_4_^2−^ and NO_3_^−^ and the lowest value of pH (Table 6). The average values of EC (2939.67), TDS (1881.39), TH (686.67), Ca^2+^ (192.67), Mg^2+^ (49.20), Cl^−^ (355.03) and NO_3_^−^(108.27) are above their respective guideline or acceptable limits of BIS^46^ and WHO^45^, except for Na^+^ (140.57), K^+^ (2.60), HCO_3_^−^ (421.00) and F^−^ (0.73), due to their elevated contents in sample numbers G17,G18 and G25 that decrease the pH in groundwater. On the other hand, only sample G18 has excess concentrations of HCO_3_^−^ and F^−^ above their acceptable limits defined by BIS^46^ (Table S2). Therefore, the specific parameters that majorly influence the C3 are EC, TDS, TH, Ca^2+^, Mg^2+^, Na^+^, K^+^, Cl^−^ and NO_3_^−^ that indicate geogenic and anthropogenic inputs enhancing the mineralization of groundwater. Finally, C2 and C3 correspond to the combination of PC1 and PC3.

## Conclusions

This paper highlights the non-carcinogenic human health risk assessment (HHRA) of NO_3_^−^ and F^−^ contamination in groundwater on four different age groups (infants, children, teens and adult) through ingestion and dermal contact using deterministic and probabilistic approaches, source apportionment of NO_3_^−^ and F^−^ with multiple parameters and chemometric modelling to extract the latent factors controlling the groundwater chemistry. Results of the deterministic and probabilistic hazard quotients ($HQ$) of nitrate ($HQ_{{{\text{NO}}_{3}^{ - } }}$) and fluoride ($HQ_{{{\text{F}}^{ - } }}$) signify that the ingestion pathway has the potential non-carcinogenic health implications on all target populations. The deterministic results of the risk certainty levels (*RCL*) of the hazard index ($HI$) above unity for nitrate ($HI_{{{\text{NO}}_{3}^{ - } }}$) stand at infants (51.85%) > children (48.15%) > teens (40.74%) = adults (40.74%) and for fluoride ($HI_{{{\text{F}}^{ - } }}$) at infants (66.67%) > children (37.04%) > adults (33.33%) > teens (25.23%). However, the probabilistically calculated *RCL* health risks in the subpopulation groups are in the order of infants ($HI_{{{\text{NO}}_{3}^{ - } }}$ = 34.03% and $HI_{{{\text{F}}^{ - } }}$ = 24.17%) > children ($HI_{{{\text{NO}}_{3}^{ - } }}$ = 23.01% and $HI_{{{\text{F}}^{ - } }}$ = 10.56%) > teens ($HI_{{{\text{NO}}_{3}^{ - } }}$ = 13.17% and $HI_{{{\text{F}}^{ - } }}$ = 2.00%) > adults ($HI_{{{\text{NO}}_{3}^{ - } }}$ = 11.62% and $HI_{{{\text{F}}^{ - } }}$ = 1.25%). These figures reveal that there exist higher degrees of potential human health risks in all the subpopulation groups in the deterministic outputs compared to those of the probabilistic model. Field observations do not support deterministic conclusions, but they do approve the probabilistic RCL values. This may be because the deterministic estimation is based on the assumption of an extreme (single point) value for all input variables individually at different concentration levels of the indicator parameters, thus possibly leading to overestimation of the output results since the extreme value may not represent the actual field conditions. Also, since the deterministic approach does not have any provision for validation of its output results, the analysis coming out of it is speculative by nature. On the contrary, the probabilistic approach provides options to choose from a range of values depending on the most likelihood field conditions besides a provision for sensitivity analysis, which enables validation of the input variables affecting the output results among the various exposure pathways. Due to all these considerations, this study concludes that probabilistic modelling is superior to deterministic approaches in human health risk assessment.

Strong positive correlation of scatter plots between NO_3_^−^ with multiple parameters (EC, TH, Ca^2+^, Mg^2+^ and Cl^−^) indicate anthropogenic inputs, such as domestic sewage, agricultural runoff, oxidation of poultry wastes, etc. Prolonged application of N fertilizers has developed cation exchange processes between NH_3_ and Ca^2+^ and Mg^2+^ enhancing the mineralization of Ca^2+^ and Mg^2+^ in groundwater, thus leading to water hardness and elevated NO_3_^−^ concentrations. The positive regression lines between F^−^ and pH, Na^+^ and HCO_3_^−^, respectively, infer that the alkaline pH with higher concentrations of NaHCO_3_ or Na^+^ or HCO_3_^−^ ions in groundwater allows dissolution of fluoride-bearing rocks, such as muscovite, biotite, amphibole, fluorite, and hornblende through rock-water interaction. Therefore, the fact that about 85% of samples show HCO_3_^−^/Ca^2+^ ratio > 1 indicates that there exist favourable groundwater conditions for further enrichment of fluoride minerals in the study area. This finding certainly shall be detrimental to the human health risks, especially of infants and children, in the long run, which is a matter of great concern for the entire study area. Chemometric modelling confirms that Ca^2+^, Mg^2+^, HCO_3_^−^, F^−^ and SO_4_^2−^ are derived from geogenic sources, Cl^−^ and NO_3_^−^ from anthropogenic inputs and Na^+^ and K^+^ from mixed factors. Further, integration of extracted principal components (PCs) with each significant cluster enables prediction of the latent parameters influencing the sampling locations and confirmation of the various sources.

The study area needs clean drinking water free from NO_3_^−^ and F^−^ for better human health. Based on the unique findings of the present work, socio-enviro conditions and hydrogeological setup, treatment of groundwater through various membrane techniques (reverse osmosis and electrodialysis), ion exchange, adsorption, coagulation, and precipitation processes are highly recommended prior to human consumption. Also, since literacy rate in the area is about 50%, effort needs to be made for mass awareness through various IEC (information, education, and communication) techniques to apprise people of the local groundwater conditions and what is best for their longevity. Further, to tackle similar problems elsewhere in the world, the evaluation of HHRA must be carried out both deterministically and probabilistically to get a holistic picture of groundwater vulnerability. Source apportionment of the contaminants too must be conducted with the help of the chemometric techniques for better human judgement.

### Supplementary Information

Supplementary Figures.

Supplementary Tables.

## Data Availability

The datasets generated during and/or analysed during the current study are already presented in the form of tables and figures in the manuscript. In case of any specific requirement, the corresponding author may please be contacted for the needful.
