# Supplementary material for: Probabilistic modelling is superior to deterministic approaches in the human health risk assessment: an example from a tribal stretch in central India
Source: Sci Rep. 2023 Nov 7;13:19351. doi: 10.1038/s41598-023-45622-1 (PMC10630383; doi:10.1038/s41598-023-45622-1)
Supplement: Supplementary file 1 — Supplementary Figures. [file 41598_2023_45622_MOESM1_ESM.docx]

**Probabilistic modelling is superior to deterministic approaches in the human health risk assessment – an example from a tribal stretch in central India**

**Rajkumar Herojeet, Rakesh K. Dewangan, Pradeep K. Naik, Janak R. Verma**

**Supplementary Information**

**List of Supplementary Figures**

Fig. S1. Plot of TDS with respect to TH concentrations.

Fig. S2. Boxplot showing relative abundance of major ions in groundwater samples.

Fig. S3. Inter-ionic relationship between major ions in groundwater: (a) Ca^2+^/Mg^2+^, (b) Na^+^ + K^+^ vs. Total Cations TZ^+^, (c) SO_4_^2-^ vs Ca^2+^, (d) Na^+^/Ca^2+^, (e) HCO_3_^-^/Ca^2+^ in the study area.


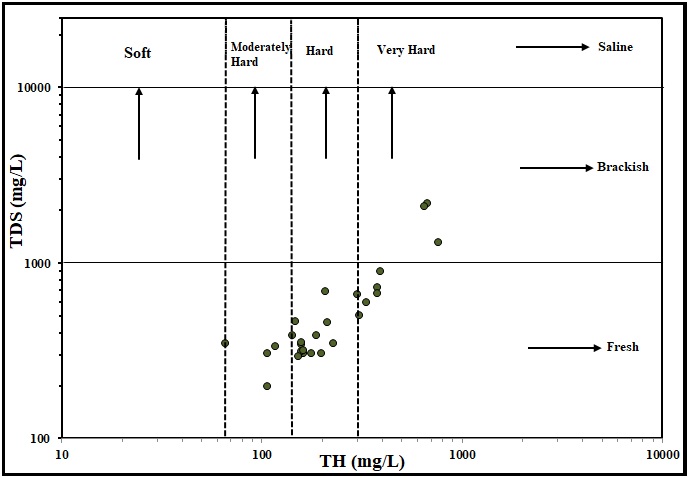


**Fig. S1. Plot of TDS with respect to TH concentrations.**

**
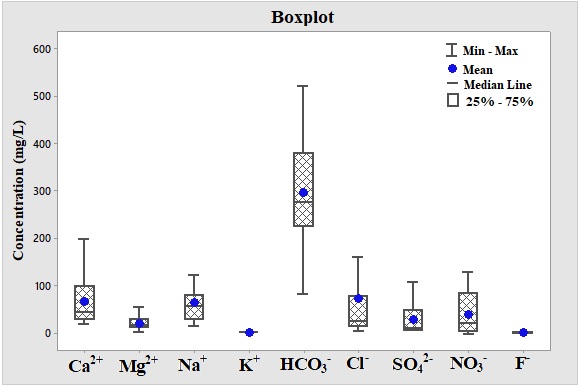
**

**Fig. S2. Boxplot showing relative abundance of major ions in groundwater samples.**

**
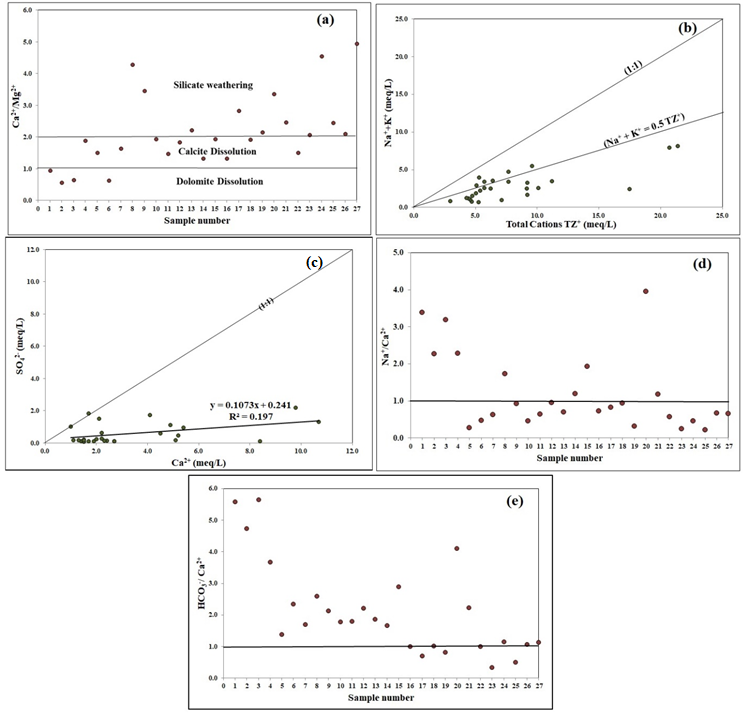
**

**Fig. S3. Inter-ionic relationship between major ions in groundwater: (a) Ca^2+^/Mg^2+^, (b) Na^+^ + K^+^ vs. Total Cations TZ^+^, (c) SO_4_^2-^ vs Ca^2+^, (d) Na^+^/Ca^2+^, (e) HCO_3_^-^/Ca^2+^ in the study area.**
