# Supplementary material for: Probabilistic modelling is superior to deterministic approaches in the human health risk assessment: an example from a tribal stretch in central India
Source: Sci Rep. 2023 Nov 7;13:19351. doi: 10.1038/s41598-023-45622-1 (PMC10630383; doi:10.1038/s41598-023-45622-1)
Supplement: Supplementary file 2 — Supplementary Tables. [file 41598_2023_45622_MOESM2_ESM.docx]

**Probabilistic modelling is superior to deterministic approaches in the human health risk assessment – an example from a tribal stretch in central India**

**Rajkumar Herojeet, Rakesh K. Dewangan, Pradeep K. Naik, Janak R. Verma**

**Supplementary Information**

**List of Supplementary Tables**

Table S1. Deterministic and probabilistic values for the exposure risks through oral and dermal pathways

Table S2. Physicochemical composition of analysed groundwater samples from village Supebeda, district Gariyaband, Chhattisgarh State, India.

Table S3. Classification of groundwater based upon EC, TDS, and TH.

Table S4. NO_3_^-^ and F^-^ concentrations and health risk assessment (after Adimalla et al. 2018).

Table S5. Statistical description of deterministically and probabilistically calculated average daily dose (*ADD)* for ingestion and dermal pathways at different age groups.

**Table S1. Deterministic and probabilistic values for the exposure risks through oral and dermal pathways.**

| Sl no. | Variables | Symbol | Units | Deterministic value | References | Distribution types | Probabilistic value | References |
| --- | --- | --- | --- | --- | --- | --- | --- | --- |
| 1 | Parameter Concentration | *C_M_* | mg/L | Metal specific |  | NO_3_^-^ (Logistic) and F^-^ (Uniform) | Metal specific |  |
| 2 | Ingestion rate of water | *IR_w_* | L/Day |  |  |  |  |  |
|  | (a) Infants | *IR I* |  | 0.65 | Zhai et al. (2017) | Lognormal | 0.61±0.27 | Mukherjee et al. (2019)^1^ |
|  | (b) Children | *IR C* |  | 1.16 | USEPA (2011) | Triangular | 1.27 (1 - 1.5) | Zhai et al. (2017)^2^; USEPA (2011)^3^ |
|  | (c) Teens | *IR T* |  | 1.98 | USEPA (2011) |  | 1.98 (1.82 - 2.34) | USEPA (2011)^3^ |
|  | (d) Adults | *IR A* |  | 2.7 | WHO (2013); USEPA (2011) |  | 2.7 (2.34 - 3.0) | WHO (2013)^4^, USEPA (2011)^3^ |
| 3 | Exposure frequency | *EF_r_* | Day/year | 350 | USEPA (1996) | Triangular | 345 (180-365) | USEPA (1996)^5^ |
| 4 | Exposure Duration | *ED* | years |  |  |  |  |  |
|  | (a) Infants | *ED I* |  | 1 | Mukherjee et al. (2019) | Uniform | 0 - 1 | Mukherjee et al. (2019)^1^ |
|  | (b) Children | *ED C* |  | 6 | Zeng et al. (2009) |  | 0 - 6 | Zeng et al. (2009)^6^; Mukherjee et al. (2019)^1^ |
|  | (c) Teens | *ED T* |  | 6 | Zeng et al. (2009) |  | 0 - 6 | Zeng et al. (2009)^6^; Mukherjee et al. (2019)^1^ |
|  | (d) Adults | *ED A* |  | 30 | USEPA (1996) |  | 0 - 30 | USEPA (1996)^5^; Mukherjee et al. (2019)^1^ |
| 5 | Exposed skin surface area | *SA* | cm2 |  |  |  |  |  |
|  | (a) Infants | *SA I* |  | 3629 | Zhai et al. (2017) | Lognormal | 3416.0± 1.18 | Mukherjee et al. (2019)^1^ |
|  | (b) Children | *SA C* |  | 7500 | USEPA (2011) |  | 7422±1.25 | Mukherjee et al. (2019)^1^ |
|  | (c) Teens | *SA T* |  | 16850 | USEPA (2011) |  | 14321±1.18 | Mukherjee et al. (2019)^1^ |
|  | (d) Adults | *SA A* |  | 19583 | USEPA (2011) |  | 18182±1.10 | Mukherjee et al. (2019)^1^ |
| 6 | Exposure Time | *ET* | h/day |  |  |  |  |  |
|  | (a) Infants | *ET I* |  | 0.54 | USEPA (2011) | Lognormal | 0.08 ± 0.0085 | Mukherjee et al. (2019)^1^ |
|  | (b) Children | *ET C* |  | 0.54 | USEPA (2011) | Lognormal | 0.13±0.0085 |  |
|  | (c) Teens | *ET T* |  | 0.54 | USEPA (2011) |  |  |  |
|  | (d) Adults | *ET A* |  | 0.71 | USEPA (2011) |  |  |  |
| 7 | Body Weight | *BW* | Kg |  |  |  |  |  |
|  | (a) Infants | *BW I* |  | 6.9 | ICMR (2010) | Triangular | 6.9 (3.25 - 9.1) | ICMR (2010)^7^; USEPA (2011^3^, 2008^8^) |
|  | (b) Children | *BW C* |  | 19 | ICMR (2010) |  | 20.71 (9.85 - 31.95) | ICMR (2010)^7^; USEPA (2011)^3^ |
|  | (c) Teens | *BW T* |  | 45 | ICMR (2010) |  | 50.11 (32.95-59.7) |  |
|  | (d) Adults | *BW A* |  | 57 | ICMR (2010) |  | 65.33 (52 - 80) |  |
| 8 | Average Time (non-carcinogenic) | *AT* | Days |  |  |  |  |  |
|  | (a) Infants | *AT I* |  | 365 | Mukherjee et al. (2019) | Fixed value | 365 | Mukherjee et al. (2019)^1^ |
|  | (b) Children | *AT C* |  | 2190 | Zeng et al. (2009) |  | 2190 | Zeng et al. (2009)^7^ |
|  | (c) Teens | *AT T* |  | 2190 | Zeng et al. (2009) |  | 2190 | Zeng et al. (2009)^7^ |
|  | (d) Adults | *AT A* |  | 10950 | USEPA (1996) |  | 10950 | USEPA (1996)^5^ |
| 9 | Dermal Permeability | *Kp* | cm/hr | 0.001 | USEPA (2011) | Fixed value | 0.001 | USEPA (2011)^3^ |
| 10 | Conversion factor | *CF* | L/cm3 | 0.001 | USEPA (2011) | Fixed value | 0.001 | USEPA (2011)^3^ |

**Table S2. Physicochemical composition of analysed groundwater samples from village Supebeda, district Gariyaband, Chhattisgarh State, India.**

| Sample nos. | Village | pH | EC | TDS | TH | Ca^2+^ | Mg^2+^ | Na^+^ | K^+^ | HCO_3_^-^ | Cl^-^ | SO_4_^2-^ | NO_3_^-^ | F^-^ |
| --- | --- | --- | --- | --- | --- | --- | --- | --- | --- | --- | --- | --- | --- | --- |
|  |  |  | µS/cm | mg/L | | | | | | | | | | |
| G1 | Sum Sagarpara | 8.1 | 737 | 471.68 | 145 | 28 | 18 | 108.9 | 0.6 | 476 | 17.8 | 5.4 | 1 | 1.9 |
| G2 | Kumdi | 8.1 | 722 | 462.08 | 210 | 30 | 32.4 | 78.3 | 2.5 | 433 | 14.2 | 10.3 | 0 | 0.7 |
| G3 | Kumhadai | 8 | 612 | 391.68 | 140 | 22 | 20.4 | 80.3 | 4.6 | 378 | 21.3 | 9.7 | 4.7 | 0.7 |
| G4 | Fulimunda | 8.2 | 528 | 337.92 | 115 | 30 | 9.6 | 78.4 | 0.6 | 336 | 7.1 | 4.8 | 1 | 1.6 |
| G5 | Nishiguda | 7.7 | 551 | 352.64 | 225 | 54 | 21.6 | 16.5 | 1.3 | 226 | 32 | 5.6 | 28.3 | 0.6 |
| G6 | Supebeda | 7.9 | 479 | 306.56 | 195 | 30 | 28.8 | 16.4 | 3.7 | 214 | 21.3 | 9 | 37.7 | 0.7 |
| G7 | Sendmuda | 7.9 | 313 | 200.32 | 105 | 26 | 9.6 | 18.6 | 1.7 | 134 | 17.8 | 9.4 | 14.1 | 0.3 |
| G8 | Sanjhakar para | 8.3 | 481 | 307.84 | 105 | 34 | 4.8 | 67.7 | 0.7 | 268 | 10.7 | 6.2 | 3.3 | 1.7 |
| G9 | Sanjhakar para | 8.2 | 539 | 344.96 | 155 | 48 | 8.4 | 51.2 | 1.3 | 311 | 10.7 | 8.3 | 3.8 | 1.4 |
| G10 | Sanjhakar para | 8 | 483 | 309.12 | 175 | 46 | 14.4 | 23.9 | 0.9 | 250 | 17.8 | 7.3 | 13.5 | 0.7 |
| G11 | Sanjhakar para | 8 | 484 | 309.76 | 160 | 38 | 15.6 | 27.8 | 1.1 | 207 | 10.7 | 6.6 | 60.4 | 0.2 |
| G12 | Badh para | 8.2 | 495 | 316.8 | 155 | 40 | 13.2 | 43.9 | 0.8 | 268.4 | 17.8 | 12.4 | 0 | 1.3 |
| G13 | Satnami para | 8 | 502 | 321.28 | 160 | 44 | 12 | 34.9 | 1.1 | 250 | 24.9 | 14 | 5.9 | 0.7 |
| G14 | Almunyia | 7.8 | 612 | 391.68 | 185 | 42 | 19.2 | 57.4 | 1.3 | 213 | 24.9 | 74 | 32.8 | 1.7 |
| G15 | Jai Thankur di ke | 8.1 | 1084 | 693.76 | 205 | 54 | 16.8 | 119.7 | 11.4 | 476 | 60.4 | 5.7 | 48.2 | 1.8 |
| G16 | Nishiguda | 7.8 | 464 | 296.96 | 150 | 34 | 15.6 | 28.5 | 1.6 | 104 | 24.9 | 89.4 | 21.6 | 0.8 |
| G17 | Nishiguda | 7.2 | 3446 | 2205.4 | 665 | 196 | 42 | 185.5 | 3.1 | 415 | 408 | 106 | 107 | 0.6 |
| G18 | Nishiguda | 7.4 | 3314 | 2121 | 640 | 168 | 52.8 | 181.4 | 2 | 519 | 380 | 5.8 | 89.2 | 1.4 |
| G19 | Fulimunda | 7.5 | 1152 | 737.28 | 375 | 102 | 28.8 | 37.6 | 2.9 | 256 | 95.9 | 9.1 | 84.4 | 0.3 |
| G20 | Chotibudi | 8.3 | 548 | 350.72 | 65 | 20 | 3.6 | 90.6 | 1.2 | 250 | 14.2 | 49.3 | 6.9 | 1.9 |
| G21 | Dahigaon | 7.7 | 554 | 354.56 | 155 | 44 | 10.8 | 59.5 | 1.2 | 299 | 10.7 | 31 | 12.1 | 1.5 |
| G22 | Model para-I | 7.7 | 1055 | 675.2 | 375 | 90 | 36 | 58.9 | 2.1 | 275 | 95.9 | 29.3 | 88.9 | 1 |
| G23 | Model para-II | 7.7 | 790 | 505.6 | 305 | 82 | 24 | 23.1 | 1.4 | 85 | 78.1 | 85 | 69.1 | 0.3 |
| G24 | Dabnai | 7.8 | 941 | 602.24 | 330 | 108 | 14.4 | 56.7 | 3 | 378 | 63.9 | 47.4 | 10.2 | 0.6 |
| G25 | Navaguda | 7.3 | 2059 | 1317.8 | 755 | 214 | 52.8 | 54.8 | 2.7 | 329 | 277 | 64.4 | 128 | 0.2 |
| G26 | Kundai Kala | 7.6 | 1413 | 904.32 | 385 | 104 | 30 | 79.2 | 3.1 | 336 | 160 | 24.1 | 96.7 | 0.7 |
| G27 | Bastipara | 7.7 | 1048 | 670.72 | 295 | 98 | 12 | 74.2 | 2.8 | 336 | 74.6 | 54 | 84.9 | 0 |

**Table S3. Classification of groundwater based upon EC, TDS, and TH.**

| **Parameters** | **Classification (mg/L)** | **Water quality** | **% of samples** |
| --- | --- | --- | --- |
| **EC**  **(CGWB and CPCB, 1999)** | <250 | Entirely safe | NIL |
|  | 250-750 | Safe practically under all conditions (moderate saline) | 62.96% |
|  | 750-2250 | Safe with permeable soils and moderate leaching (Medium to high saline) | 26.63% |
|  | 2250-4000 | Used on soils with good permeability and with special leaching for salt tolerant crops (high saline) | 7.41% |
|  | 4000-6000 | Used only on highly permeable soils with frequent leaching with plants of high salt tolerance (very high salinity) | NIL |
|  | >6000 | This class represents water that is unfit for irrigation (excessive salinity) | NIL |
| **TDS**  **(Davies and DeWiest, 1967)** | <500 | Desirable for drinking | 62.96% |
|  | 500-1000 | Permissible for drinking | 25.93% |
|  | 1000-3000 | Useful for irrigation | 11.11% |
|  | >3000 | Unfit for drinking and irrigation | NIL |
| **TDS**  **(Freeze and Cherry, 1979)** | <1000 | Freshwater | 88.89% |
|  | 1000-10000 | Brackish Water | 11.11% |
|  | 10,000-100,000 | Saline Water | NIL |
|  | >100,000 | Brine water | NIL |
| **TH**  **(Sawyer and McCarty, 1967)** | <75 | Soft | 3.70% |
|  | 75-150 | Moderately hard | 22.22% |
|  | 150-300 | Hard | 44.44% |
|  | >300 | Very hard | 29.63% |

**Table S4. NO_3_^-^and F^-^ concentrations and health risk assessment**

**(after Adimalla et al. 2018^9^).**

| **Parameters** | **Health risk (mg/L)** | **% of samples** |
| --- | --- | --- |
| **NO_3_^-^** | No risk:< 45 | 62.96% |
|  | High risk: 45- 100 | 29.63% |
|  | Very High risk: >100 | 7.41% |
| **F^-^** | Dental caries (<0.5) | 22.22% |
|  | Required level for human health (0.6- 1.5) | 55.56% |
|  | Dental Fluorosis (1.6 – 2.0) | 22.22% |
|  | Dental and skeletal Fluorosis (2.1 – 3.0) | NIL |
|  | Leads to skeletal Fluorosis ( >3.0) | NIL |

**Table S5. Statistical description of deterministically and probabilistically calculated average daily dose (*ADD)* for ingestion and dermal pathways at different age groups.**

| Premonsoon | Parameter | Deterministic value (Ingestion) | | | | | Probabilistic value (Ingestion) | | | | |
| --- | --- | --- | --- | --- | --- | --- | --- | --- | --- | --- | --- |
| Age Group |  | Mean | Median | Stdev. | 5^th^ percentile | 95^th^ percentile | Mean | Median | Stdev. | 5^th^ percentile | 95^th^ percentile |
| Infants | NO_3_^-^ | 3.53E+00 | 1.95E+00 | 3.61E+00 | 2.71E-02 | 9.41E+00 | 1.45E+00 | 8.43E-01 | 3.99E+00 | -1.19E+00 | 5.98E+00 |
| Children |  | 2.29E+00 | 1.26E+00 | 2.34E+00 | 1.76E-02 | 6.10E+00 | 8.76E-01 | 5.63E-01 | 2.32E+00 | -8.86E-01 | 3.58E+00 |
| Teens |  | 1.65E+00 | 9.11E+00 | 1.69E+00 | 1.27E-02 | 4.39E+00 | 6.14E-01 | 4.01E-01 | 1.62E+00 | -6.27E-01 | 2.46E+00 |
| Adults |  | 1.77E+00 | 9.81E+00 | 1.82E+00 | 1.36E-02 | 4.73E+00 | 5.63E-01 | 3.88E-01 | 1.48E+00 | -5.96E-01 | 2.31E+00 |
| Infants | F^-^ | 8.46E-02 | 6.32E-02 | 5.30E-02 | 1.81E-02 | 1.69E-01 | 4.39E-02 | 2.95E-02 | 9.24E-02 | 2.49E-03 | 1.34E-01 |
| Children |  | 5.49E-02 | 4.10E-02 | 3.44E-02 | 1.17E-02 | 1.09E-01 | 2,81E-02 | 2.12E-02 | 5.37E-02 | 1.87E-03 | 7.82E-02 |
| Teens |  | 3.95E-02 | 2.95E-02 | 2.48E-02 | 8.44E-03 | 7.89E-02 | 1.72E-02 | 1.22E-02 | 3.32E-02 | 5.41E-04 | 5.01E-02 |
| Adults |  | 4.26E-02 | 3.18E-02 | 2.67E-02 | 9.08E-03 | 8.63E-02 | 1.65E-02 | 1.21E-02 | 3.16E-02 | 5.45E-04 | 4.71E-02 |
|  | Parameter | Deterministic value (Dermal) | | | | | Probabilistic value (Dermal) | | | | |
| Infants | NO_3_^-^ | 1.06E-02 | 5.88E-03 | 1.09E-02 | 8.17E-05 | 2.84E-02 | 6.54E-04 | 4.21E-04 | 1.70E-03 | -5.82E-04 | 2.60E-02 |
| Children |  | 7.98E-03 | 4.41E-03 | 8.18E-03 | 6.13E-05 | 2.13E-02 | 7.27E-04 | 4.70E-04 | 1.90E-03 | -6.33E-04 | 2.83E-03 |
| Teens |  | 7.57E-03 | 4.19E-03 | 7.76E-03 | 5.82E-05 | 2.02E-02 | 5.66E-04 | 3.81E-04 | 1.47E-03 | -5.31E-04 | 2.24E-03 |
| Adults |  | 9.13E-03 | 5.05E-03 | 9.36E-03 | 7.02E-05 | 2.44E-02 | 5.06E-04 | 3.42E-04 | 1.31E-03 | -5.14E-04 | 2.02E-03 |
| Infants | F^-^ | 2.55E-04 | 1.91E-04 | 1.60E-04 | 5.45E-05 | 5.09E-04 | 1.98E-05 | 1.48E-05 | 3.80E-05 | 1.43E-06 | 5.49E-05 |
| Children |  | 1.92E-04 | 1.43E-04 | 1.20E-04 | 4.09E-05 | 3.82E-04 | 2.18E-05 | 1.60E-05 | 4.22E-05 | 1.45E-06 | 6.12E-05 |
| Teens |  | 1.82E-04 | 1.36E-04 | 1.14E-04 | 3.88E-05 | 3.63E-04 | 1.58E-05 | 1.16E-05 | 3.02E-05 | 5.82E-07 | 4.51E-05 |
| Adults |  | 2.19E-04 | 1.64E-04 | 1.37E-04 | 4.68E-05 | 4.37E-04 | 1.45E-05 | 1.08E-05 | 2.77E-05 | 4.48E-07 | 4.13E-05 |

**References**

1. Mukherjee, I., Singh, U.K., & Patra, P.K. Exploring a multi-exposure-pathway approach to assess human health risk associated with groundwater fluoride exposure in the semi-arid region of east India. *Chemos.* **233**:164–173 (2019). [https://doi.org/10.1016/j.chemosphere.2019.05.278](https://doi.org/10.1016/j.chemosphere.2019.05.2788)
2. Zhai, Y., Lei, Y., Wu, J., Teng, Y., Wang, J., Zhao, X., & Pan, X. Does the groundwater nitrate pollution in China pose a risk to human health? A critical review of published data. *Environ. Sci. Pollut. Res.* **24**: 3640–3653 (2017). <https://doi.org/10.1007/s11356-016-8088-9>
3. USEPA (U.S. Environmental Protection Agency). Exposure factors handbook ed (Final), http://cfpub.epa.gov/ncea/risk/recordisplay.cfm?deidD236252, US Environmental Protection Agency, Washington DC (2011).
4. WHO (World Health Organization). Updated WHO/WEDC Technical Notes on WASH in Emergencies “Technical notes on drinking water, sanitation and hygiene in emergencies” World Health Organization / Water Engineering Development Centre (2013).
5. USEPA (U.S. Environmental Protection Agency). Quantitative Uncertainty Analysis of Superfund Residential Risk Pathway Models for Soil and Groundwater: White Paper. US Environmental Protection Agency, USA (1996).
6. Zeng, G., Liang, J., Guo, S., Shi, L., Xiang, L., Li, X., & Du, C. Spatial analysis of human health risk associated with ingesting manganese in Huangxing Town, Middle China. *Chemos.* **77**: 368-375 (2009). <https://doi.org/10.1016/j.chemosphere.2009.07.020>
7. ICMR (Indian Council of Medical Research). Nutrient requirements and recommended dietary allowances for Indians. A report of the expert group of the Indian Council of Medical Research. Hyderabad: National Institute of Nutrition (2010).
8. USEPA (U.S. Environmental Protection Agency). User’s Guide: Human Health Risk Assessment; United States Environmental Protection Agency: Washington, DC, USA (2008).
9. Adimalla, N., Li, P., & Venkatayogi, S. Hydrogeochemical Evaluation of Groundwater Quality for Drinking and Irrigation Purposes and Integrated Interpretation with Water Quality Index Studies. *Environ. Process.* **5**:363–383 (2018). https://doi.org/10.1007/s40710-018-0297-4
